# Effects of New P2X7R Antagonists on Retinal Inflammatory Degenerative Conditions

**DOI:** 10.1007/s10753-026-02513-7

**Published:** 2026-04-25

**Authors:** Chiara Bianca Maria Platania, Federica Conti, Maria Consiglia Trotta, Nicoletta Marchesi, Sonia Panico, Francesca Lazzara, Caterina Claudia Lepre, Marina Russo, Carlo Gesualdo, Francesca Simonelli, Michele D’Amico, Filippo Drago, Alessia Pascale, Settimio Rossi, Valeria Tarallo, Claudio Bucolo

**Affiliations:** 1 Department of Biomedical and Biotechnological Sciences, University of Catania, Catania, Italy; 2 Center for Research in Ocular Pharmacology-CERFO, University of Catania, Catania, Italy; 3 Department of Experimental Medicine, University of Campania “Luigi Vanvitelli”, Naples, Italy; 4 Department of Drug Sciences, Section of Pharmacology, University of Pavia, Pavia, Italy; 5 Institute of Genetics and Biophysics ‘Adriano Buzzati-Traverso’ - CNR, Naples, Italy; 6 Department of Medicine and Surgery, “Kore” University of Enna, 94100 Enna, Italy; 7 Department of Mental, Physical Health and Preventive Medicine, University of Campania “Luigi Vanvitelli”, Naples, Italy; 8 Multidisciplinary, Department of Medical, Surgical and Dental Sciences, University of Campania “Luigi Vanvitelli”, Naples, Italy; 9 Department of Life Sciences, Health and Health Professions, Link Campus University, 00165 Rome, Italy

**Keywords:** P2X7 receptor antagonists, Inflammation, Inflammasome, Retinal degeneration, Retina

## Abstract

Purinergic receptor (P2X7R), an adenosine triphosphate (ATP) gated nonselective cation channel, is an emerging target for several neurodegenerative conditions, including retinal degeneration. In particular, P2X7R has an established role in multiple inflammatory and immune responses, being implicated in the formation and activation of NLRP3 inflammasome leading to inflammatory priming and release of immune cytokines. Despite several efforts in P2X7R antagonist design, no drugs have reached the market yet. In this perspective, through an integrated in silico and in vitro approach, we aimed to repurpose FDA approved drugs in search of novel safe treatments for retinal degenerative diseases, where P2X7R has been found to play a detrimental role. Virtual screening of about 10,000 FDA approved drugs identified 6 putative P2X7 antagonists. Three out of these six compounds inhibited hP2X7, but not rat receptors, while only one compound, tipranavir, inhibited also mouse P2X7R. Then, for these three P2X7R antagonists, namely bazedoxifene, teniposide and tipranavir, we have evaluated the in vitro tolerability by treating immortalized human Müller cells (MIO-M1), primary retinal endothelial cells (HRECs), human umbilical vein endothelial cells (HUVECs) and human retinal pigmented epithelial cells (hRPEs). Tipranavir and teniposide were well tolerated by the mentioned retinal cells, thereby they have been further evaluated in in vitro models of glaucoma, diabetic retinopathy, and age-related macular degeneration. Tipranavir and teniposide showed retinal cell protection, modulating the expression of HIF-1α, VEGF-A, and inflammatory cytokines. Therefore, these compounds are worthy of further investigation to manage retinal degenerative conditions.

**Supplementary Information:**

The online version contains supplementary material available at 10.1007/s10753-026-02513-7.

## Introduction

 The purinergic P2X7 receptor (P2X7R ), an adenosine triphosphate (ATP) gated nonselective cation channel, is an emerging target for several neurodegenerative and proliferative diseases. Indeed, as recently reviewed by Platania et al. (2022), several preclinical in vitro and in vivo studies evidenced that P2X7R is an intriguing pharmacological target for treatment of retinal diseases, such as glaucoma, diabetic retinopathy (DR) and age-related macular degeneration (AMD) [1]. New data, specifically on preclinical and clinical studies, has further supported the hypothesis of P2X7R role in the pathogenesis of glaucoma [2], DR [3] and AMD [4], and generally in retinal degenerations, including retinal dystrophies [5].

P2X7R activation is particularly significant in the context of acute and chronic inflammation. After retinal injury, activated immune cells produce inflammatory mediators and ATP, this latter binds with P2X7R, leading to receptor activation and amplification of inflammatory response [6]. An over- or dysregulated activation of P2X7R is linked to chronic inflammation and neuronal cell death. Indeed, P2X7R activation is a potent stimulus to elicit several cellular mediators, such as Nuclear factor kappa-light-chain-enhancer of activated B cells (NF-κB), Hypoxia-inducible factor 1-alpha (HIF-1α), and Phosphoinositide 3-kinase/Protein Kinase B/Glycogen Synthase Kinase-3 beta (PI3K-AKT-GSK-3β) pathways, along with the nucleotide-binding domain, leucine-rich repeat, and pyrin domain-containing protein 3 (NLRP3) inflammasome activation [7].

Currently, several efforts in medicinal chemistry are ongoing for the development of selective and potent P2X7R antagonists [8–11]. However, as highlighted by recent reviews, clinical development of P2X7R antagonists is affected by attrition factors that are intrinsic to receptor structure and function [12]. In fact, at inflammatory sites, the extracellular ATP concentration is higher than in healthy sites [12], therefore negative allosteric modulators might not effectively inhibit P2X7R-mediated responses. Indeed, as evidenced by P2 × 7RP2X7R structural data from the paper by Karasawa and Kawate (2016), there is a steric hindrance for antagonists at an allosteric site, upon binding with ATP [13].

Recently, McCarthy et al. (2019) resolved the full-length structure of the rat P2X7R, which bears a calyx glass shape, where receptor extracellular side corresponds to the bowl, the intramembrane region would be the stalk, and the intracellular bundle endowed with guanosine triphosphatase (GTPase) activity resembles the base [14]. McCarthy underlined that palmitoylation of P2X7R is necessary for the inhibition of receptor desensitization, upon stimulation with ATP. Indeed, P2X7R is characterized by an intrinsic structural complexity, which complicates the design and development of ligands.

However, there are several successful examples of drug repurposing preclinical studies focusing on P2X7, such as the antiviral zidovudine, which ameliorated the pathologic signs in a model of Duchenne dystrophy [15], and the uricosuric drug probenecid that inhibited inflammation in a severe influenza A virus mouse model [16].

In this perspective, the aim of our study is a drug repurposing campaign in search of Food and Drug Administration (FDA) approved drugs with an activity as P2 × 7R antagonists and a therapeutic potential in glaucoma, DR and AMD, through an integration of high throughput virtual screening approaches and different in vitro models of retinal diseases.

For this purpose, we first used the full length apo (closed state) structure of the rat receptor to model the human P2X7R, as previously reported [17]. Thereby, we carried out a virtual screening campaign within ~ 10,000 FDA approved drugs, at the allosteric pocket of P2X7R as evidenced by Karasawa and Kawate [13].

The binding of top scored compounds was then tested on mouse, rat, and human P2X7R. Three compounds inhibited human receptor (IC50 micromolar range), namely bazedoxifene, teniposide and tipranavir, a selective estrogen receptor modulator, a podophyllotoxin derivative, and a protease inhibitor, respectively.

Particularly, bazedoxifene, a selective estrogen receptor modulator, is currently approved, to manage vasomotor symptoms associated with menopause and to prevent postmenopausal osteoporosis [18]; while teniposide is a chemotherapeutic agent, specifically a topoisomerase II inhibitor, used to treat several forms of solid tumors, leukemia and lymphoma [19]. Lastly, tipranavir, a nonpeptidic antiretroviral protease inhibitor, is employed in the therapy and prevention of human immunodeficiency virus (HIV) infection [20]. Noteworthy, bazedoxifene and teniposide evidenced anti-inflammatory and neuroprotective effect [21–24]. Moreover, teniposide is clinically tested to manage retinoblastoma [25, 26]. Additionally, several HIV protease inhibitors showed anti-angiogenic activity [27, 28].

Based on these premises, we settled in vitro models of glaucoma, DR, and AMD, designed to deliver inflammatory and pro-angiogenic stimuli, according to previous reported studies and further investigated the three mentioned molecules. Particularly, different in vitro models of retinal degeneration have been used to resemble the main pathological features involved in glaucoma, DR, and AMD diseases. Additionally, this would strengthen the translational value of the present study.

Specifically, the in vitro model of glaucoma involved human immortalized Moorfields Institute of Ophthalmology-Müller 1 cells (MIO-M1 cells) subjected to hypoxic damage [29, 30]. Concerning DR, we tested tipranavir, teniposide and bazedoxifene in primary human retinal endothelial cells (HRECs) exposed to high glucose levels [31]. Additionally, the antiangiogenic potential of the repurposed drugs has been evaluated on human umbilical vein cord endothelial cells (HUVECs) challenged with phorbol 12-myristate 13-acetate (PMA) [32]. Finally, an in vitro AMD paradigm was exploited using human immortalized retinal pigmented epithelial (hTERT RPE-1) cells treated with *Alu* RNA able to activate the NLRP3 inflammasome, through the P2X7R signaling [33, 34].

The three repurposed drugs have been tested for their tolerability on the mentioned retinal cell types, but only tipranavir and teniposide have been well tolerated by Müller, endothelial and retinal pigmented epithelial cells. These two repurposed P2X7R antagonists, protected retinal cells from hypoxic damage and inflammatory stimuli. Additionally, tipranavir and teniposide, attenuated expression of Vascular Endothelial Growth Factor-A (VEGF-A) and inhibited angiogenesis in endothelial cells.

## Materials and Methods

### In Silico Approaches

Virtual screening of FDA approved drugs has been carried out on our previous published human model of full-length P2X7R [17]. Specifically, we used the consensus model based on the x-ray structure of full-length rat receptor (PDB: 6U9V). The Grid for virtual screening was centered at pocket of P2X7R allosteric antagonists identified with the Sitemap^®^ task of Schrodinger Maestro, as shown in our previous published paper [17]. At first, sdf files of FDA approved drugs have been downloaded (access December 2022) at the server https://www.bindingdb.org/bind/ByFDAdrugs.jsp. Thereafter 3D structural data of FDA approved drugs have been generated with the ligand preparation task. Virtual screening task of Schrodinger © maestro was characterized by three docking steps with increasing precision methods. Glide HTVS (flexible docking with penalization of non-planar amide bonds; post docking minimization with strain correction terms, 5 poses per compound, keep of 30% of best compounds, retention of all states); Glide SP (enhanced sampling, flexible docking with penalization of non-planar amide bonds; post docking minimization with strain correction terms, grid constrains, 3 poses per compound, keep of 20% of best compounds, retention of all good states), and Glide XP (enhanced sampling, flexible docking with penalization of non-planar amide bonds; post docking minimization with strain correction terms, grid constrains, 2 poses per compound, keep of 10% of best compounds, retention of only best states). The predicted ΔG_binding_ was used as a rescoring approach of the virtual screening, as automatically calculated after the last docking step (Glide XP), which indeed included MM-GBSA calculation [6].

### P2X7R Ligand Binding Assays

The compounds (Table 1, Merk Darmstadt, Germany or TOCRIS, Bristol, UK) were dissolved in dimethyl sulfoxide (DMSO, Sigma Aldrich, Milan, Italy) at the required assay concentration prior to diluting in assays buffer; the final dimethyl sulfoxide (DMSO) concentration did not exceed 0.5%. A438079 (TOCRIS, Bristol, UK) has been used as a reference compound for all the functional assays (from 100 mM, serial dilution 1:10).

**Table 1:** List of the compounds tested in binding assays

Compound name	Top conc (µM)	Dilution	Steps	Merck code
Zidovudine	10	1:5	8	S2579
Teniposide	10	1:5	8	SML0609
Atracurium besylate	10	1:5	8	PHR2482
Etoposide	10	1:5	8	E1383
Nelfinavir mesylate hydrate	10	1:5	8	PZ0013
Tipranavir	10	1:5	8	SML2312
Aprepitant	10	1:5	8	SML2215
Bazedoxifene acetate	10	1:5	8	PZ0018
Compound name	**Top conc (µM)**	**Dilution**	**Steps**	**Tocris code**
A438079	100	1:10	8	2972

Human embryonic kidney 293 (HEK293, ATCC CRL-1573) cells were seeded at 5–8 × 10^6^ cells in a T225 flask, growth till confluence in EMEM, supplemented with 10% Fetal Bovine Serum (FBS), 2 mM Glutamine Stable, 1x Penicillin/Streptomycin (P/S) medium and split every 2–3 days. Cells were stably transfected with Biorad Gene Pulser to express mP2 × 7R (pcDNA3.1_mP2RX7, electroporation), rP2 × 7R (pcDNA3.1_rP2RX7, electroporation) and hP2 × 7R (pcDNA3.1_hP2RX7, electroporation).

#### Fluo-8 NW Calcium Assay

HEK293-P2RX7 cells were seeded in 384 Well Polystyrene Assay Plates, black/clear bottom, PDL (Greiner Bio-One S.r.l, Roma, Italy) at 20,000 cells per well. 24 h after seeding, cells were incubated with 20 ml/well of Screen Quest™ Fluo-8 NW Calcium Assay Kit 0.5X in Assay Buffer for 1 h at room temperature (RT) (AAT Bioquest, California, USA). Buffer composition: Hanks’ Balanced Salt Solution (HBSS) 1x; NaHCO_3_ 4.17 mM; HEPES 20 mM (pH 7.4), sterile filtered. Then 10 µl/well of test compounds and controls at 3X concentrated in Assay Buffer + Pluronic-68 0.005% were injected at the Thermo Scientific™ Varioskan™ (ThermoFisher, Waltham, Massachusetts, United States) and the Kinetic response was recorded over a period of 2 min (min). The DMSO final concentration was 0.5%. After 20 min, a second injection of 15 µl/well of 3X concentrated reference agonist (at ~EC80) in Assay buffer + Pluronic-68 0.005% + Bovine serum albumin (BSA) 0.0003% final concentration was performed, and the signal of the emitted fluorescence was recorded for additional 2 min. Tips pre-primed in Assay Buffer with BSA 0.0003% final concentration were used. A pre-plate was tested to calculate the agonist EC80. Fluorescence was recorded and normalized versus Positive Controls (BzATP EC80) and Inhibitor Controls (A438079 IC100) to obtain the Activity [%] for each well.

#### YO- PRO Assays

HEK293-P2RX7 cells were seeded in 384 Well Polystyrene Assay Plates, black/clear bottom, PDL (Greiner Bio-One S.r.l, Roma, Italy) at 20,000 cells per well. 24 h after seeding the medium was removed and the cells were washed with Low Divalent Cation Assay Buffer. Assay buffer consists of Tyrode’s buffer low Calcium (0.3 mM Ca^++^) Magnesium free, where standard Tyrode’s buffer composition is NaCl 130 mM, KCl 5 mM, CaCl_2_ 2 mM, MgCl_2_ 1 mM, NaHCO_3_ 5 mM, HEPES 20 mM; pH 7.4. The cells were pre-loaded with 20 µl/w of 5 µM YO-PRO dye (Y3603, ThermoFisher, Waltham, Massachusetts, United States) and fluorescence measurement immediately started. Then 10 ml/w of test compounds or reference antagonist A438079 at 3X-concentration were injected with the Thermo Scientific™ Varioskan™ (ThermoFisher) and the kinetic response was monitored over a period of three minutes (Exc wavelength 470–495 nm, Em wavelength 515–575 nm). After 20 min, a second injection of 10 ml/w of 4X reference activator (BzATP, B6396 Sigma-Aldrich Milan, Italy, at EC80 in Assay Buffer + BSA 0.0003% final concentration) was performed and the signal of the emitted fluorescence was recorded for additional sixty min. The effect of the test compounds was reported as percent inhibition versus the reference antagonist at saturating concentration and IC50 values were calculated accordingly.

### In Vitro Models

Human MIO-M1 cells subjected to hypoxia stimulus, primary HRECs challenged with high glucose, HUVECs treated with PMA and hTERT RPE-1 cells treated with *Alu* RNA have been employed as in vitro models of glaucoma, diabetic retinopathy and age-related macular degeneration, respectively. Four different in vitro models of retinal degenerative diseases have been employed to strengthen the translational value of results.

#### Drugs Treatment

Human MIO-M1 cells, HRECs, HUVECs, and hTERT RPE-1 cells have been treated with P2 × 7R antagonists, such as teniposide (Teni, SML0609, Sigma-Aldrich, St. Louis, Missouri, United States), tipranavir (Tipra, SLM2312, Sigma-Aldrich, St. Louis, Missouri, United States) and bazedoxifene (Baze, PZ0018, Sigma-Aldrich, St. Louis, Missouri, United States) at concentrations of 1,10, and 100 µM, chosen on the basis of P2 × 7R IC50 (Table 3), to assess cell tolerability. After, 1 and 10 µM concentrations of Teni and Tipra have been selected for the subsequent experiments. Whereas, Baze has been excluded, based on cytotoxicity results (Table 5). In all experiments, cells were pre-treated with Tipra and Teni for 1 h and the challenged with different stimuli.

#### Müller Cell Culture and Hypoxia Damage

Human MIO-M1 cells were purchased from the Institute of Ophthalmology, University College of London (UCL) (RRID: CVCL_0433). MIO-M1 were cultured at 37 °C (humidified atmosphere with 5% carbon dioxide - CO_2_) in GlutaMAXTM DMEM medium (31966-021, Gibco, ThermoFisher, Waltham, Massachusetts, United States) with 100 U/mL P, 100 µg/ml S, and 10% FBS. After reaching confluence (∼70%), MIO-M1 were used for experimental procedures. Particularly, optimal cell density was obtained by seeding 1.5 × 10^4^ cells/well in 96-well plates (Costar, Corning, New York). After 24 h of culture, MIO-M1 were treated with Teni, Tipra and Baze (1–10-100 µM) in medium containing FBS 10% for 24 h, in normoxic conditions. At the end of the treatment, cell viability has been assessed through the Cell Counting Kit-8 (CCK-8) assay.

As regard as hypoxia damage model, MIO-M1 were exposed to low percentage of oxygen (~ 1%) by means of hypoxia incubator chamber (27310, Stemcell technologies, Vancouver, Canada) to induce hypoxic damage. Particularly, 1.5 × 10^4^ or 5 × 10^5^ cells were seeded in 96-well plates (Costar, Corning, New York) or 60 mm petri dishes (Costar, Corning, New York), respectively. After 24 h of culture, cells were starved with 0.1% FBS for 18 h. After that, cells were pre-treated with Teni and Tipra (1–10-100 µM) for 1 h in normoxic condition and then were placed in the hypoxia incubator chamber for 7 h. After hypoxia exposure, cells were subjected to cytotoxicity assays (CCK-8, LDH and ATPlite assay) and western blot analysis. As regards western blot, MIO-M1 were cultured in 60 mm petri dishes at a density of 5 × 10^5^. MIO-M1 cells cultured in normoxic conditions were used as control.

#### Primary HRECs and High Glucose Treatment

Primary HRECs (cell passage #4–6), purchased from Innoprot (Elexalde Derio, Spain), were cultured with endothelial cell medium (ECM, P60104 Innoprot; Elexalde Derio, Spain, RRID: CVCL_B5WJ) supplemented with 5% FBS, 1% endothelial cell growth supplement (ECGS), 1% of P/S solution. Cells were grown with a normal glucose concentration (5 mM) at 37 °C, with a humidified atmosphere of 5% CO_2_. Particularly, optimal cell density was obtained by seeding 1 × 10^4^ HRECs/well were seeded in 96-well plates (Costar, Corning, New York). After 24 h of culture, HRECs were treated with Teni, Tipra and Baze (1–10-100 µM) for 48 h. At the end of the treatment, cell viability has been assessed through CCK-8 assay.

As regards high glucose challenge, 1 × 10^4^ HRECs/well were seeded in 96-well plates (Costar, Corning, New York), and after 24 h of culture, HRECs were serum-starved for 24 h with 1% FBS. After that, cells were pre-treated with Tipra and Teni (1 and 10 µM) for 1 h and then incubated for 48 h with high glucose (HG, 25 mM) [35–37]. Control cells were cultured in normal glucose (Ctrl, 5 mM). Whereas 25 mM of mannitol (M) was used as an osmotic control. Cell viability (CCK-8 assay) and VEGF-A levels in culture medium have been assessed.

#### HUVECs and PMA Model

HUVECs were obtained from Sigma-Aldrich (Milan, Italy), plated in 25 cm^2^ flasks, and cultured in an all-in-one ready-to-use medium (Endothelial Cell Growth Medium; Sigma-Aldrich, Milan, Italy). The flasks were incubated at 37 °C in a humidified atmosphere containing 5% CO_2_. Particularly, 3.5 × 10^5^ cells/well were seeded into 96-well plates (Costar, Corning, New York). After, 48 h of treatment with Teni, Tipra and Baze (1–10-100 µM), cell viability has been assessed through 3-[4,5-dimethylthiazol-2-yl]−2,5-diphenyl tetrasodium bromide (MTT) assay.

As regard as PMA model, HUVECs were seeded at a density of 100.000 cell/well. After 24 h of culture, cells were pre-treated with Tipra and Teni (1 and 10 µM) for 1 h and then incubated for 48 h with PMA (p8139, Sigma-Aldrich, Milan, Italy) at 100 nM (positive control). At the end of the treatment, VEGF levels have been measured through Western Blot analysis. Additionally, tube formation assay has been carried out (Supplementary materials).

#### Human Immortalized Retinal Pigmented Epithelial Cell Culture and Alu RNA Model

hTERT RPE-1 cells were purchased from ATCC^®^ (CRL-4000, RRID: CVCL_4388) and cultured in Dulbecco’s modified Eagle’s medium medium F12 (DMEM F/12) supplemented with 10% FBS, 0.01 mg/ml Hygromycin B (Thermo Scientific), 2 mM glutamine and standard concentration of antibiotics (P/S, EuroClone, Milan, Italy).

hTERT RPE-1 cells were seeded at a density of 6.200 cells/cm^2^. After 24 h of culture, cells were treated with Teni, Tipra and Baze (1–10-100 µM) for 24 h. Cell viability has been assessed through CellTiter 96 AQueous One Solution Cell Proliferation Assay (Promega, Madison, WI, USA).

As regards *Alu* RNA model, we synthesized a 281 nucleotides *Alu* RNA sequence originating from the CDNA clone TS 103, which is known to be expressed in human cells [38]. The pT7/*Alu* plasmid was linearized with DraI and subjected to HiScribe T7 Quick High Yield RNA Synthesis Kit (BioLabs, Ipswich, MA, USA) according to the manufacturer’s instructions. After the T7 transcription reaction, RNA was treated with DNase and purified following phenol–chloroform extraction and ethanol precipitation. RNA was quantified at Nanodrop, and the integrity was monitored by gel electrophoresis. This yields single stranded RNA that is folded into a defined secondary structure identical to Pol III derived transcripts.

Cells were pre-treated for 1 h at 37 °C with drugs (Tipra and Teni 1 and 10 µM) or vehicle and then transfected with *Alu* RNA or Mock (vehicle, 0.5%DMSO, Sigma-Aldrich, Milan) using Lipofectamine 2000 (Invitrogen, Carlsbad, CA, USA) according to the manufacturer’s instructions. After *Alu* RNA transfection, cell viability (through MTT assay), NLRP3 and interleukin (IL-18) mRNA expression, as well as caspase-1 activity have been assessed.

### Cytotoxicity Assays

The MTT assay (Chemicon, Temecula, CA) was carried out on HUVECs, HRECs, and MIO-M1 cells to assess cell viability, according to manufacturer’s instructions. Particularly, after treatments as described above, cells were first incubated at 37 °C with MTT (5 mg/ml) for 2 h; then DMSO 100 µl per well were added and absorbance was measured at 570 nm in a plate reader (VariosKan, Thermo Fisher Scientific, Waltham, MA). Results were expressed as % of control.

hTERT RPE-1 cell viability was assessed using CellTiter 96 AQueous One Solution Cell Proliferation Assay (Promega, Madison, WI, USA) according to manufacturer’s instructions. This assay is based on the reduction of 3-(4,5-dimethylthiazol-2-yl)−5-(3-carboxymethoxyphenyl)−2-(4-sulfophenyl)−2 H-tetrazolium (MTS) to a soluble formazan by NAD(P)H-dependent enzymes in metabolically active cells. Viability is expressed as a percentage relative to non-transfected “Mock” cells (set at 100%). Mock represents the cells ‘empty’ transfected with Lipofectamine 2000; these cells showed slightly reduced viability compared to untreated cells, reflecting the minor inherent toxicity of the transfection reagent.

Lactate dehydrogenase (LDH) cell release was measured using the CyQUANT™ LDH Cytotoxicity assay (C20301, ThermoFisher, Waltham, Massachusetts, United States) on MIO-M1 cells. After treatment as described above, according to the manufacturer’s protocol, lysis solution was added to positive control wells (non-treated cells) for 45 min. After transferring 50 µL of the medium into a new multi-well, 50 µL of the reaction mixture was added. After 30 min at room temperature, 50 µL of the stop solution was added lastly. The absorbance values were measured at 490 nm using a plate reader (VarioSkan, Thermo Fisher Scientific, Waltham, MA). LDH release was normalized to control. Moreover, absorbance values were edited by subtracting medium absorbance.

Cell viability was further assessed on MIO-M1 cells by measurement of ATP production by means of the PerkinElmer ATPlite 1step Luminescence Assay System according to the manufacturer’s protocol (6016731, PerkinElmer, Connecticut, United States). After seeding and treatments with P2 × 7R antagonists as described above, MIO-M1 cells were washed twice with Phosphate-buffered saline (PBS) 1X, and 100 µL of buffer solution (ATPlite) was added to each well, according to the manufacturer’s protocol. After 2 min of incubation at RT (shaker, 700 rpm), luminescence was assessed using the Varioskan microplate reader (VarioSkan, Thermo Fisher Scientific, Waltham, MA). Results were reported as percentage of control.

### Western Blot Analysis

HIF-1α and VEGF-A protein levels have been investigated on MIO-M1 and HUVEC cell lines, respectively.

As regards MIO-M1 cells, after treatments described above, proteins from cell lysates were extracted with RIPA Buffer, including protease and phosphatase inhibitors cocktail (Sigma-Aldrich, St. Louis, MO, USA). Total protein content, in each cell lysate sample, was determined by the BCA Assay Kit (23227, Pierce™ BCA Protein Assay Kit, Invitrogen, Life Technologies, Carlsbad, CA, USA). Extracted proteins (20 µg) were loaded on the NuPAGE TM 10% Bis-Tris gel (NP0315BOX, ThermoFisher, Waltham, Massachusetts, United States). After electrophoresis, proteins were transferred into a nitrocellulose membrane (1620115, Bio-rad, California, United States). Membranes were blocked with 5% milk in Tris-buffered saline 0.2% Tween 20 (TBST) for 1 h at RT. Therefore, membranes were incubated overnight (4 °C) with appropriate primary anti-HIF-1α (Mouse, NB100-105, Novus Biologicals, Colorado, United States, 1:200 dilution) and anti- beta actin (β-actin) (Rabbit, A2066-100UL, Sigma-Aldrich, St. Louis, Missouri, United States, 1:500 dilution) antibodies. After overnight incubation, the membranes were then incubated with secondary chemiluminescent antibodies (ECL anti-mouse, NA931 and ECL anti-rabbit, NA934, Cytiva Amersham, United Kingdom, 1:2000 dilution) for 1 h at RT. After secondary antibodies, membranes were incubated with enhanced chemiluminescence (ECL) (SuperSignal™ West Pico PLUS Chemiluminescent Substrate, 34,577, Thermo Fisher Scientific, Carlsbad, CA, United States) and were detected through I-BrightTM 1500 (A43679, Invitrogen, Life Technologies, Carlsbad, CA, United States) by chemiluminescence. Densitometry analyses of blots were performed at non-saturating exposures and analyzed by ImageJ software (NIH, Bethesda, MD). The values were normalized to β-actin, which was used as housekeeping control.

As regard as HUVEC cells, proteins were measured according to Bradford’s method using bovine serum albumin as an internal standard. Proteins were diluted in 2x Sodium Dodecyl Sulphate (SDS) protein gel loading solution, boiled for 5 min, and separated into 12% SDS-PAGE. The anti-VEGF rabbit monoclonal antibody (Abcam, Ab52917, Cambridge, MA, United States) and the monoclonal α-Tubulin (Sigma-Aldrich, MA1-80017) were both diluted at 1:1000, based on each data sheet instructions. Concerning the specific Western blotting procedure, we followed the protocol published in our previous paper [39]. Densitometric analyses were performed using the ImageJ (https://imagej.net/ij/) image-processing program. The values were normalized to α-Tubulin which was used as housekeeping control.

### Enzyme-Linked Immunosorbent Assay (ELISA)

VEGF-A levels were measured in culture medium of HRECs cells, after treatments as described above, using a commercially available ELISA kit according to the manufacturer’s protocols (ABIN411369 antibodies-online Inc.; Limerick, PA, USA).

### Quantitative Reverse Transcription Polymerase Chain Reaction (qPCR)

NLRP3 and IL-18 mRNA expression have been assessed on hTERT RPE-1 cells, after treatments as described above.

In particular, total RNA was isolated using Trizol reagent (Invitrogen, Carlsbad, CA, USA), DNase treated and reverse transcribed (QuantiTect, QIAGEN, Hilden, Germany) into cDNAs. This was amplified by real-time quantitative PCR on CFX Opus 96 Real-Time PCR System (BioRad, Hercules, CA, USA) with SYBR green Master Mix (iTaq Universal SYBR Green Supermix, Biorad, cat. number 1725124). Oligonucleotide primers specific for human NLRP3 (forward 5’-GCACCTGTTGTGCAATCTGAA-3’ and reverse 5’-TCCTGACAACATGCTGATGTGA-3’); human interleukin 18 (IL-18) (forward 5’-ATCACTTGCACTCCGGAGGTA-3’ and reverse 5’-AGAGCGCAATGGTGCAATC-3’) and human β-Actin (forward 5′-CTCTTCCAGCCTTCCTTCCT-3′ and reverse 5′-TGTTGGCGTACAGGTCTTTG-3′) were used. Primer sequences were designed and order at Integrated DNA Technologies (IDT). The qPCR cycling conditions were 50 °C for 2 min and 95 °C for 10 min, followed by 40 cycles of a two-step amplification program (95 °C for 15 s and 58 °C for 1 min). At the end of the amplification, melting curve analysis was performed using the dissociation protocol from the Sequence Detection system to exclude contamination with nonspecific PCR products. The PCR products were also confirmed by agarose gel electrophoresis, which revealed only one specific band of the predicted size. For negative controls, no cDNAs were used as templates in the qPCR, and the results were verified by the absence of bands in the gel. The relative expression of the target genes was determined by the 2^–ΔΔCt^ method. Analysis and presentation of PCR and RT-PCR results adhered to MIQE guidelines [40].

### Caspase-1 Activation Assay

Caspase-1 activity was measured on hTERT RPE-1 cells, after treatments as described above, using the Cell Meter Live Cell Caspase-1 Binding Assay Kit - Green Fluorescence - (AAT Bioquest, Inc., cat. number 20108) according to the manufacturer’s instructions. After completion of staining, data was acquired with FACSCanto (BD Biosciences).

### Tube-Formation Assay

To confirm the anti-angiogenic activity of tested molecules (Teni and Tipra), tube-formation assay using Matrigel was carried out. HUVEC cells (1.5 × 10^4^ cells/well) were seeded into pre-coated with Matrigel (CLS356234, Corning^®^ Matrigel^®^ Basement Membrane Matrix) 96-well plate. The following experimental groups were included in the assay: control, 100 nM PMA, Teni (1 µM) and Tiprar (1 µM) + PMA. The 96-well plate was incubated for 4 h at 37 °C, 5% CO_2_, 95% humidity. Images were collected with Leica DMi8 (widefield inverted microscope). Total branching point and the mean loop area (px) of total neo-vessel network were measured using WimTube (https://www.wimasis.com/en/WimTube, Wimasis GmbH, Munich, Germany).

### Data Analysis and Statistics

Data analysis is reported as % effect in relation to the normalization standards and curve fitting is performed with GraphPad Prism software.

The Kinetic Response Value (KRV) is calculated as follows:

KRV = Delta between the Max kinetic peak computed from Sect. 5 to the end of the kinetic minus the pre-injection calculated as mean relative functional unit (RFU) from Sect. 3 to Sect. 4 of the kinetic. The resulting value is then divided by the baseline calculated as mean RFU from Sect. 1 to Sect. 2.

$$\:\frac{Max\:\left(sec5-end\right)-Mean\:(sec3-sec4)\:}{Mean\:(sec1-sec2)}\:$$

The KRV is normalized versus Positive Controls and Inhibitor Controls in order to obtain the Activity [%] for each well. Normalization places the compound activity values on an equivalent scale and makes them comparable across plates or different compound batches. Therefore, the compound activity values are scaled (based on the two references) to a common range (two-point normalization).

The final equation to calculate the Activity% can be simplified as follow

$$\:\%\:Activity=\:-100\frac{x-<PositiveControls>}{<PositiveControls>-<InhibitorControls>}$$

Where:

- x is the calculated signal value of a well (RV).
- < PositiveControls > is the median of the calculated signal values (RV) for the Central Reference wells of a plate (median of Positive Controls).
- < InhibitorControls > is the median of the calculated signal values (RV) for the Scale Reference wells of a plate (median of Inhibitor Controls).
- full inhibition corresponds to %Activity = −100.

The fitting of the dose-response curve of each test compound is performed with GraphPad Prism software on the normalized values and applying the following fitting equation:

$$\:Y=S0+\frac{Sinf-S0}{1+{\left(\frac{{10}^{LogAC50}}{{10}^{x}}\right)}^{n}}$$

where X is Log_10_ of compound concentration.

The equation has four parameters:

1. Zero Activity (S0) - Activity level at zero concentration of test compound.
2. Infinite Activity (Sinf) - Activity level at infinite concentration of test compound.
3. AC50 - Concentration at which activity reaches 50% of maximum level. This term corresponds to IC50 in this assay.
4. Hill coefficient (n) - Measure of the slope at AC_50_.

Data analysis was carried out by investigators unaware of experimental group labels. Graph design and statistics has been done by two investigators, before labels were unveiled by another investigator. A power analysis, setting as p-value cut-off < 0.05, was carried out to choose sample size. We carried out *n* = 4 independent in vitro experiments, and data are expressed as mean ± SD (*n* = 4). The Shapiro-Wilk test was used to test the normal distribution of data. Thereby, we carried out one-way ANOVA, followed by Tukey-Kramer post-hoc test for multiple comparisons between groups. Post-hoc test was carried out given an F with *p* < 0.05, and no significant variance inhomogeneity was found within groups. Differences between groups were considered significant at *p* < 0.05. The following software were used for statistical analysis: SPSS software, version 21.0 (SPSS Inc., Chicago, IL, USA), and GraphPad Prism version 10 (San Diego, CA, USA). The software GraphPad Prism version 10 has been used also for graph design.

## Results

###  High Throughput Virtual Screening of FDA Approved Drugs in Search of P2X7R Antagonists

**Fig. 1:**
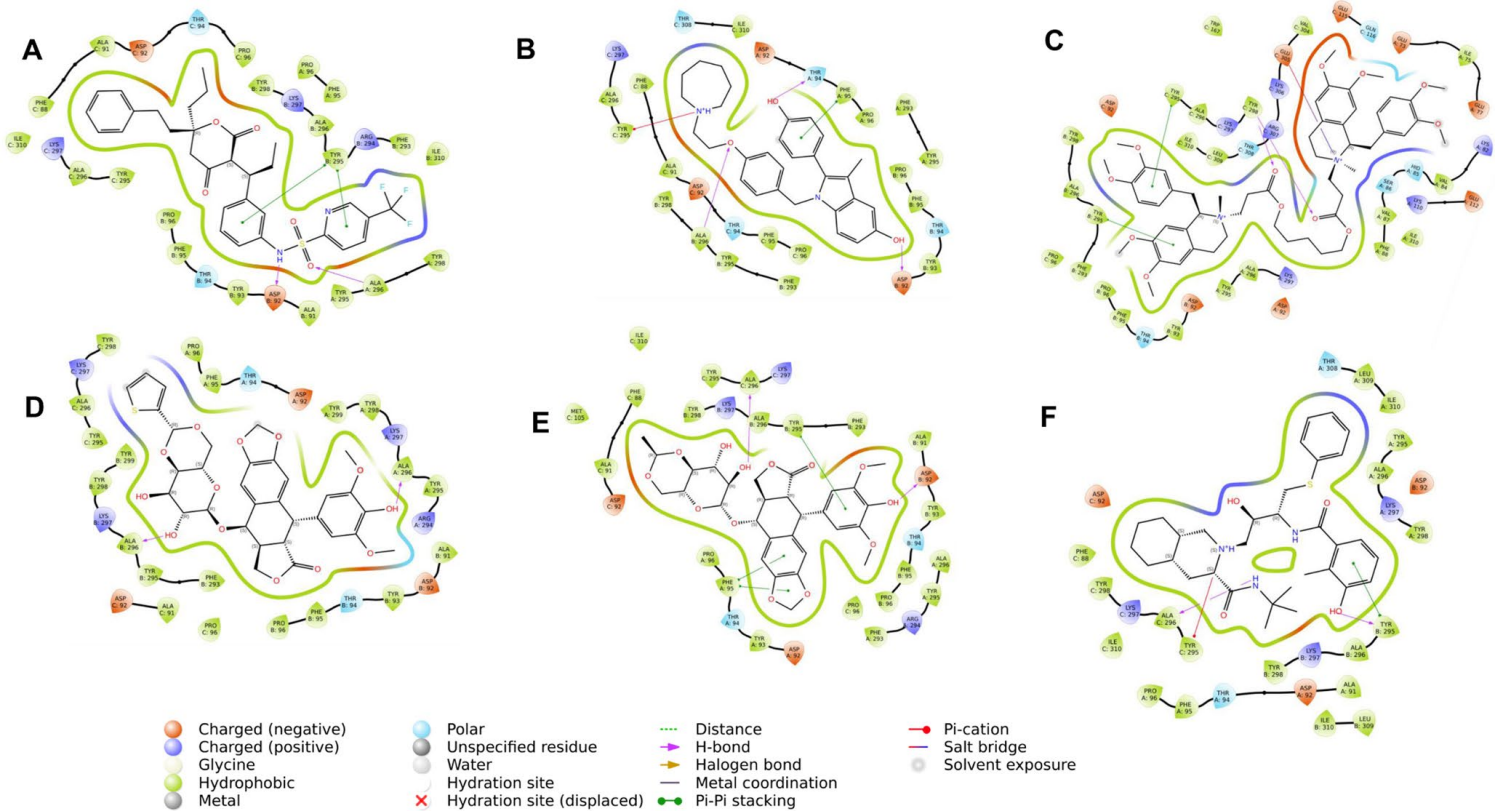
Ligand – hP2X7 receptor interactions representation. (**A**) tipranavir; (**B**) bazedoxifene; (**C**) atracurium besylate; (**D**) teniposide; (**E**) etoposide; (**F**) nelfinavir bound to hP2 × 7 receptor at allosteric site typically occupied by selective allosteric modulators, such as JNJ47965567 (PDB: 5U1X) [13] and A438079 [41]

The virtual screening of ~ 10,000 compounds on the full length of P2X7R human model (template PDB: 6U9V, closed P2 × 7 state) has been carried out with automatic sequential application of different molecular docking steps: Glide HTVS, Glide SP, Glide XP, having each step increasing precision of molecular interaction modeling. Only the molecules that reached the Glide XP step have been automatically rescored with MMGBSA calculations (Table 2). Within the compounds that reached the final virtual screening step (Glide XP), tipranavir (Tipra), bazedoxifene (Baze), atracurium besylated and teniposide (Teni) have shown the lowest (most favorable) predicted ΔG_binding_. Besides several antiviral drugs have been retrieved with the virtual screening as top scored ligands for P2X7R (Table 2), zidovudine did not pass any virtual screening step, which preceded the MMGBSA calculations. Figure 1 shows 2D representation of ligand-protein interactions for top scored ligands after virtual screening at allosteric binding site of our model of the full-length hP2X7R, after MMGBSA calculation and refinement, all ligands preferentially interact through hydrophobic interactions with residues of the three chains (A, B, C) of hP2X7R channel.

**Table 2:** Virtual screening results – MMGBSA ΔG_binding_

Compound	MMGBSA ΔG_binding_ on full-length hP2X7R model (PDB 6U9V) (Kcal/mol)
tipranavir	−94,73
bazedoxifene	−93,7
atracurium besylate	−90,84
teniposide	−90,08
etoposide	−75,07
nelfinavir	−48,61
zidovudine	not scored

### Ligand Binding of Top Scored Compounds on Murine and Human P2X7 Receptor

To confirm the virtual screening results, the compounds have been functionally tested at human, mice and rat P2X7R expressed in HEK-293 cells. We included zidovudine based on previous findings, as mentioned in the introduction. The Fluo-8 NW Calcium assay (Table 3) showed that within the six top scored compounds retrieved with the virtual screening, only Teni, Tipra and Baze have been found to be active as antagonists at hP2X7R, while only atracurium besylate and Tipra have been found to be active as antagonists at mouse P2X7 receptor. None of the tested compounds were found to modulate rat channel activity.

**Table 3:** Fluo-8 NW calcium assay. Compounds activity on hP2X7R, mP2X7R and rP2X7R was tested and compared to BzATP (positive control) and A438079 (inhibitor control)

Compound	Human agonist	Mouse agonist	Rat agonist	Human antagonist	Mouse antagonist	Rat antagonist
Ligand	**pIC50**	**pIC50**	**pIC50**	**pIC50**	**pIC50**	**pIC50**
zidovudine	inactive	inactive	inactive	inactive	inactive	Inactive
teniposide	inactive	inactive	inactive	5.44	inactive	Inactive
atracurium besylate	inactive	inactive	inactive	inactive	4.83	Inactive
etoposide	inactive	inactive	inactive	inactive	inactive	Inactive
nelfinavir mesylate hydrate	inactive	inactive	inactive	inactive	inactive	Inactive
tipranavir	inactive	inactive	inactive	5.04	5.03	Inactive
bazedoxifene acetate	inactive	inactive	inactive	5.36	inactive	Inactive

We confirmed with the YOPRO assay the micromolar activity of Teni (IC50 1.45 µM), Tipra (IC50 13.5 µM), and Baze (IC50 1.26 µM) as hP2X7R antagonists (Table 4).

**Table 4:** YOPRO assay. Compounds activity on hP2X7R was tested and compared to BzATP (positive control) and A438079 (inhibitor control)

Compound Info	Human P2X7R_YO-PRO
Ligand	**pIC50**
zidovudine	inactive
teniposide	5.84
atracurium besylate	inactive
etoposide	inactive
nelfinavir mesylate hydrate	inactive
tipranavir	4.87
bazedoxifene acetate	5.90

###  Tipra, Teni and Baze Cytotoxicity Assays on MIO-M1, HRECs, HUVECs and hRPEs Cells

We carried out cytotoxicity assays to test retinal cell viability of Teni, Tipra and Baze with concentrations ranging from 1 to 100 µM (Table 5). This concentration range has been chosen based on the experimental IC50 of these compounds as antagonists at hP2X7R (Tables 3 and 4).

**Table 5:** Tipranavir, teniposide and bazedoxifene cytotoxicity in retinal cells. # MTS assay for hRPE cells. Other cell viability tests were carried out with the MTT assay. N.S. stands for non-significant modification in cell viability compared to control at all tested concentrations (1–10-100 µM). * *p* < 0.05 vs. Ctrl

Compound	Muller (% control)	HRECs (% control)	HUVECs (% control)	hRPE (% control)
Tipranavir	N.S.	40 ± 3 (100 µM)*	10 ± 2 (100 µM)*	25 ± 3 (100 µM)*
Teniposide	60 ± 4 (100 µM)*	35 ± 8 (100 µM)*	45 ± 3 (100 µM)*	50 ± 3 (100 µM)*
Bazedoxifene	20 ± 2 (10 µM)* 15 ± 2 (100 µM)*	34 ± 6 (10 µM)* 20 ± 3 (100 µM)*	75 ± 2 (10 µM)* 45 ± 3 (100 µM)*	24 ± 2 (100 µM)*

Müller MIO-M1 cells tolerated treatment with Tipra (24 h exposure) in basal conditions from 1 to 100 µM concentration, while Teni at 100 µM concentration decreased cell viability. Baze decreased significantly MIO-M1 cell viability at 10 and 100 µM concentrations after 24 h exposure (*p* > 0.05 vs. Ctrl). In HRECs exposed to normal glucose (5 mM; Ctrl) cell viability was not affected by 10 µM Tipra (*p* > 0.05 vs. Ctrl) and Teni (*p* > 0.05 vs. Ctrl). Conversely, cell viability was significantly reduced by 10 µM Baze (*p* < 0.05 vs. Ctrl). All the three compounds (after 48 h of exposure) significantly decreased HREC cell viability at 100 µM concentration (*p* < 0.05 vs. Ctrl). As regards HUVECs, we tested the cell viability up to 48 h. As shown in Tables 5 and 10µM Teni, Tipra and Baze were not toxic to cells treated for 48 h (decrease in cell viability < 70% vs. control group [42]).

Then, we tested whether Teni, Tipra and Baze could affect hRPEs cell viability, after 24 h of exposure. As shown in Table 5, the drugs exhibited toxicity when used at the highest concentration (100µM) (*p* > 0.05 vs. Ctrl), while they were not cytotoxic at both 1µM and 10µM concentration. We excluded further experiments, hereby reported, Baze due to its greater toxicity in MIO-M1 cells, HRECs and HUVECs, compared to Tipra and Teni.

### Hypoxia-Induced Cytotoxicity on MIO-M1 Cells

**Fig. 2:**
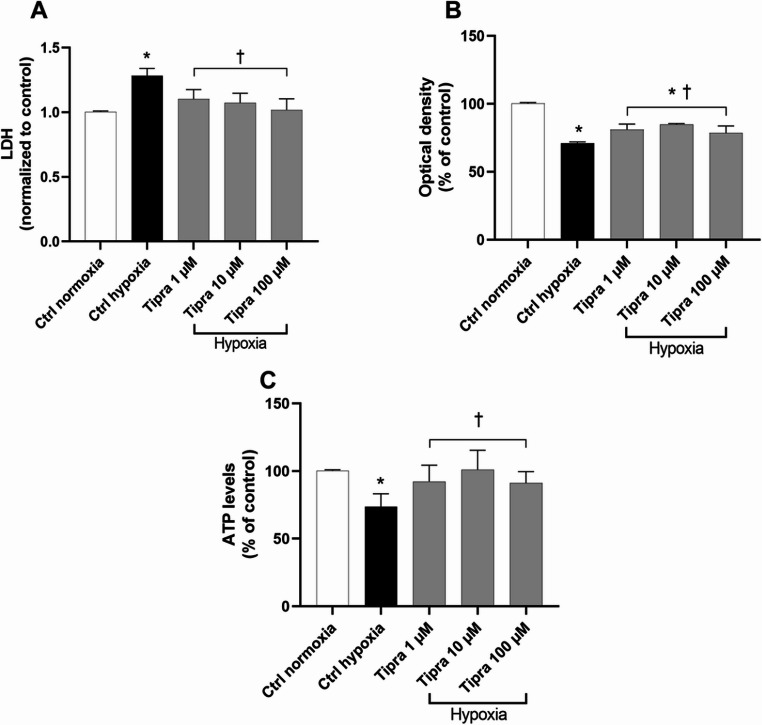
Tipranavir protected MIO-M1 cells exposed to hypoxia. MIO-M1 cells were pre-treated in normoxic condition for 1 h with tipranavir (Tipra, 1–10–100 µM) and subsequently exposed to hypoxia insult for 7 h. After this time, LDH (**A**), CCK-8 (**B**) and ATPlite (**C**) assays were carried out to evaluate cytotoxicity. Values are reported as mean ± SD (*n* = 4). Data were analyzed by one-way ANOVA and the Tukey *post hoc* test for multiple comparisons. *p < 0.05 vs. ctrl normoxia; †*p* < 0.05 vs. ctrl hypoxia

**Fig. 3:**
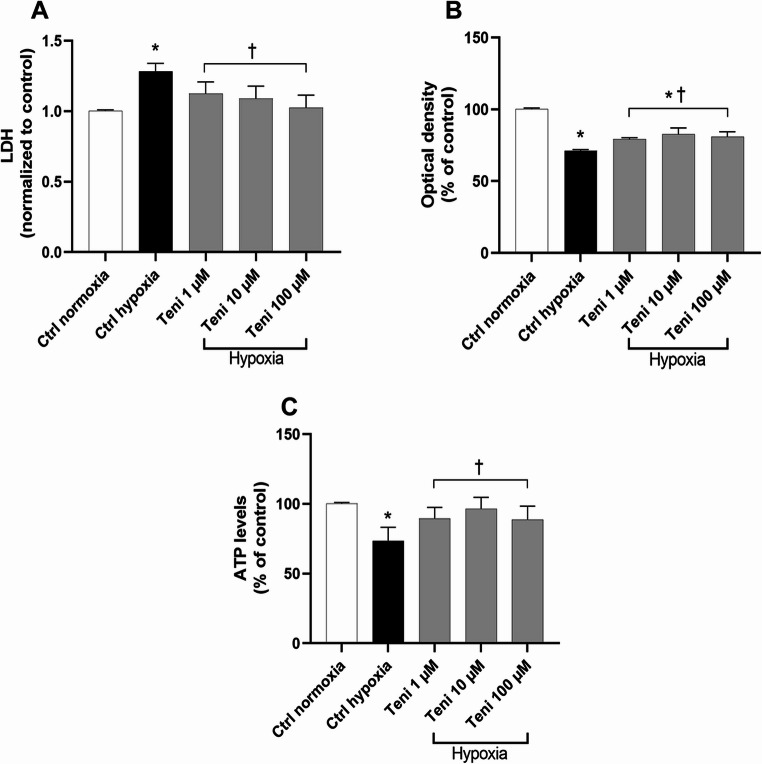
Teniposide reduced LDH release and increased cell viability and ATP levels after hypoxia insult. MIO-M1 cells were pre-treated in normoxic condition for 1 h with teniposide (Teni, 1–10–100 µM) and then exposed to hypoxia damage for 7 h. After this time, LDH (**A**), CCK-8 (**B**) and ATPlite (**C**) assays were carried out to assess cytotoxicity. Values are reported as mean ± SD (*n* = 4). Data were analyzed by one-way ANOVA and the Tukey *post hoc* test for multiple comparisons. *p < 0.05 vs. ctrl normoxia; †*p* < 0.05 vs. ctrl hypoxia

As shown in Figs. 2e and 3, the hypoxic stimulus led to a significant (*p* < 0.05) reduction of cell viability and ATP levels, along with a significant (*p* < 0.05) increase of LDH release in MIO-M1, in comparison to control cells kept in normoxic environment. Tipra and Teni, at all tested concentrations (1–10-100 µM), were able to significantly counteract (*p* < 0.05) the hypoxia-induced cytotoxicity in MIO-M1 cells. Indeed, these compounds increased cell viability, along with ATP levels, and reduced LDH release compared to MIO-M1 cells only exposed to hypoxic injury (Figs. 2 and 3).

Although safe to MIO-M1 cells, we did not test 100 µM Tipra and Teni in further experiments, because this concentration has shown toxic effects in other cell type.

### HIF-1α Protein Expression

**Fig. 4:**
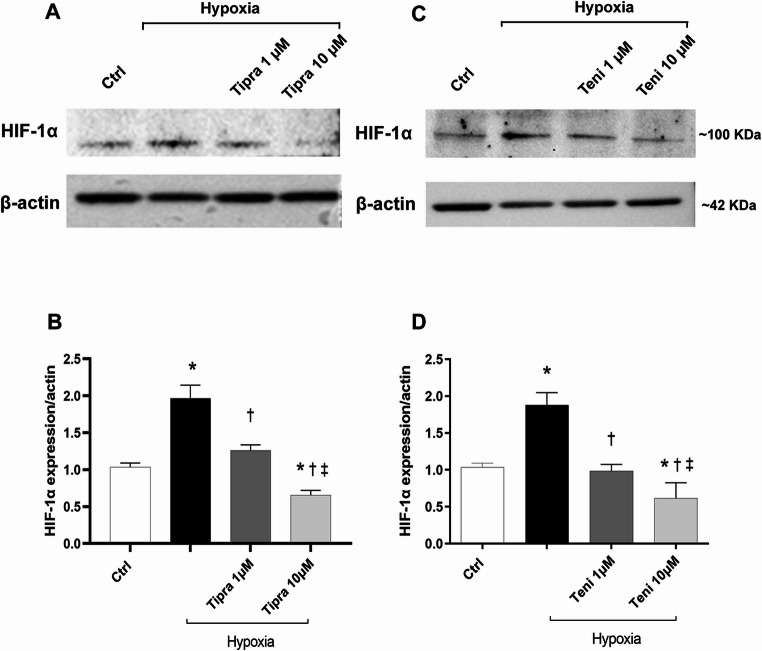
Tipranavir and teniposide counteracted hypoxia-induced HIF-1α expression. MIO-M1 cells were pre-treated in normoxic condition for 1 h with tipranavir (Tipra; 1 and 10 µM, **A** and **B**) and teniposide (Teni,1 and 10 µM, **C** and **D**) and then exposed to hypoxia insult for 7 h. (**A**, **B**) Representative immunoblots and densitometric analysis of HIF-1α in lysates of MIO-M1 treated with tipranavir. (**C**, **D**) Representative immunoblots and densitometric analysis of HIF-1α in lysates of MIO-M1 treated with teniposide. Densitometry analysis of each band was carried out with the Image J program; HIF-1α expression has been normalized to β-actin values. Values are reported as mean ± SD (*n* = 4). Data were analyzed by one-way ANOVA and the Tukey *post hoc* test for multiple comparisons. *p < 0.05 vs. ctrl; †*p* < 0.05 vs. hypoxia; ‡*p* < 0.05 vs. Tipra 1 µM (B) and vs. Teni 1 µM (D)

As shown in Fig. 4 7 hours of hypoxia insult led to a significant increase (*p* < 0.05) of HIF-1α expression compared to control cells (normoxic condition). Indeed, both Tipra and Teni, at the selected concentrations of 1 and 10 µM, significantly (*p* < 0.05) reduced HIF-1α expression in a concentration dependent manner, compared to hypoxia-exposed MIO-M1 cells, showing a protective effect (Fig. 4) against the hypoxic insult.

### HREC Cell Viability and VEGF-A Levels After Exposure to High Glucose

**Fig. 5:**
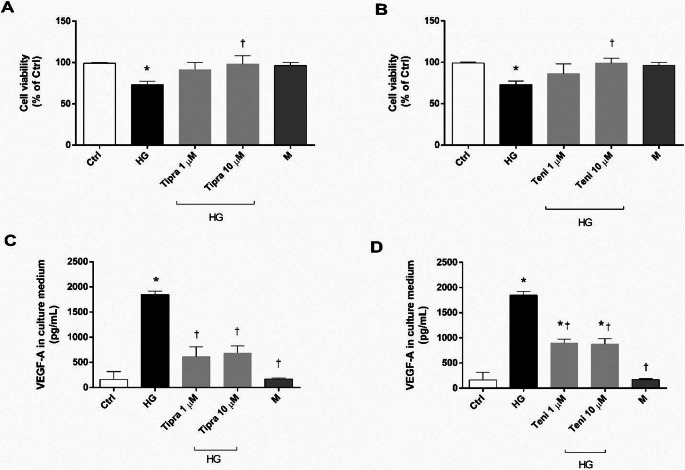
Tipranavir and teniposide improved cell viability and reduced VEGF-A secretion in HRECs exposed to high glucose concentration. HRECs cells were pre-treated for 1 h with tipranavir (Tipra; 1 and 10 µM, **A** and **C**) and teniposide (Teni,1 and 10 µM, **B** and **D**) and then challenged with high glucose (25 mM, HG) for 48 h. Control cells were cultured in normal glucose (5 mM, Ctrl), mannitol (25 mM) was used as osmotic control. After 48 h, CCK-8 (**A**, **B**) and ELISA assays (**C**, **D**) were performed to evaluate respectively cell viability and secreted VEGF-A levels. Values are reported as mean ± SD (*n* = 4). Data were analyzed by one-way ANOVA and the Tukey *post hoc* test for multiple comparisons. *p < 0.05 vs. Ctrl; †*p* < 0.05 vs. HG

As expected, the exposure of HRECs to high glucose (25 mM; HG) markedly reduced cell viability (*p* < 0.05 vs. Ctrl). This was significantly restored in HRECs treated with both Tipra and Teni 10 µM (*p* < 0.05) (Fig. 5A, B).

Accordingly, VEGF-A levels in HRECs culture medium were not modified in mannitol treated group (M) compared to Ctrl, while VEGF-A levels markedly increased by HG exposure (*p* < 0.05 vs. Ctrl). Tipra and Teni treatments (1–10 µM) significantly reduced VEGF-A levels in HRECs exposed to HG (*p* < 0.05) (Fig. 5C, D).

### HUVEC Cell Viability and VEGF-A Levels After Exposure to PMA

**Fig. 6:**
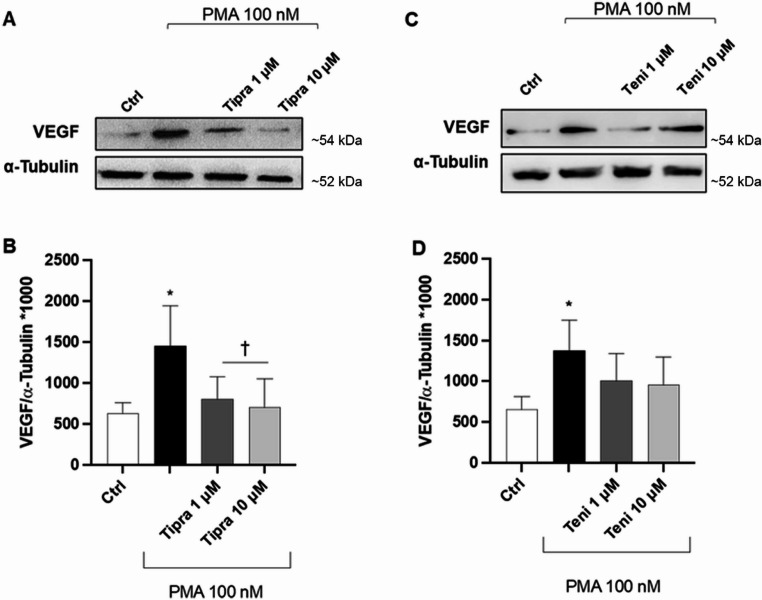
Tipranavir and teniposide protected HUVECs against PMA-induced VEGF-A release. HUVECs cells were pre-treated for 1 h with tipranavir (Tipra; 1 and 10 µM, **A** and **B**) and teniposide (Teni,1 and 10 µM, **C** and **D**) and then challenged for 48 h with phorbol 12-myristate 13-acetate (PMA; 100nM) alone or in the presence of tipranavir (Tipra; A, B) or teniposide (Teni; C, D) at 1µM or 10 µM. Control cells were treated for 48 h with the solvent (dimethyl sulfoxide; Ctrl). Densitometry analysis of each band was carried out with the Image J program; VEGF expression has been normalized to α-Tubulin value. Values are reported as mean ± SD (*n* = 4). Data were analyzed by one-way ANOVA and the Tukey *post hoc* test for multiple comparisons. *p < 0.05 vs. ctrl; †*p* < 0.05 vs. PMA

HUVECs were exposed to PMA, which mimics diacylglycerol (DAG, the physiologic activator of protein kinase C), whose synthesis is triggered by hyperglycemia. As previously demonstrated, PMA at 100nM induces an intracellular increase of VEGF-A in HUVECs following 48 h of treatment [43]. Therefore, we evaluated the combined effect of PMA (100 nM) and Tipra or Teni (1 and 10 µM) on VEGF protein levels in HUVECs following 48 h of exposure. The data showed in Fig. 6, indicate the capability of both compounds, at 1 µM and 10 µM concentrations, to counteract the increase of VEGF protein content induced by PMA treatment; although only Tipra treatment led to a significant reduction of VEGF levels (*p* < 0.05), compared to positive control cells (Fig. 6A, B). To confirm the anti-angiogenic activity of this two repurposed P2X7R antagonists (Teni and Tipra), tube-formation assay with Matrigel was carried out. As reported in Fig. 1S (supplementary materials), Tipra and Teni (1 µM) were able to significantly (*p* < 0.05) inhibit angiogenesis induced by the PMA. Angiogenesis has been quantified as total branching point and mean loop area (px) of total neo-vessel network (Fig. 1S).

###  Protection from Alu RNA-Induced Inflammation in hRPEs

**Fig. 7:**
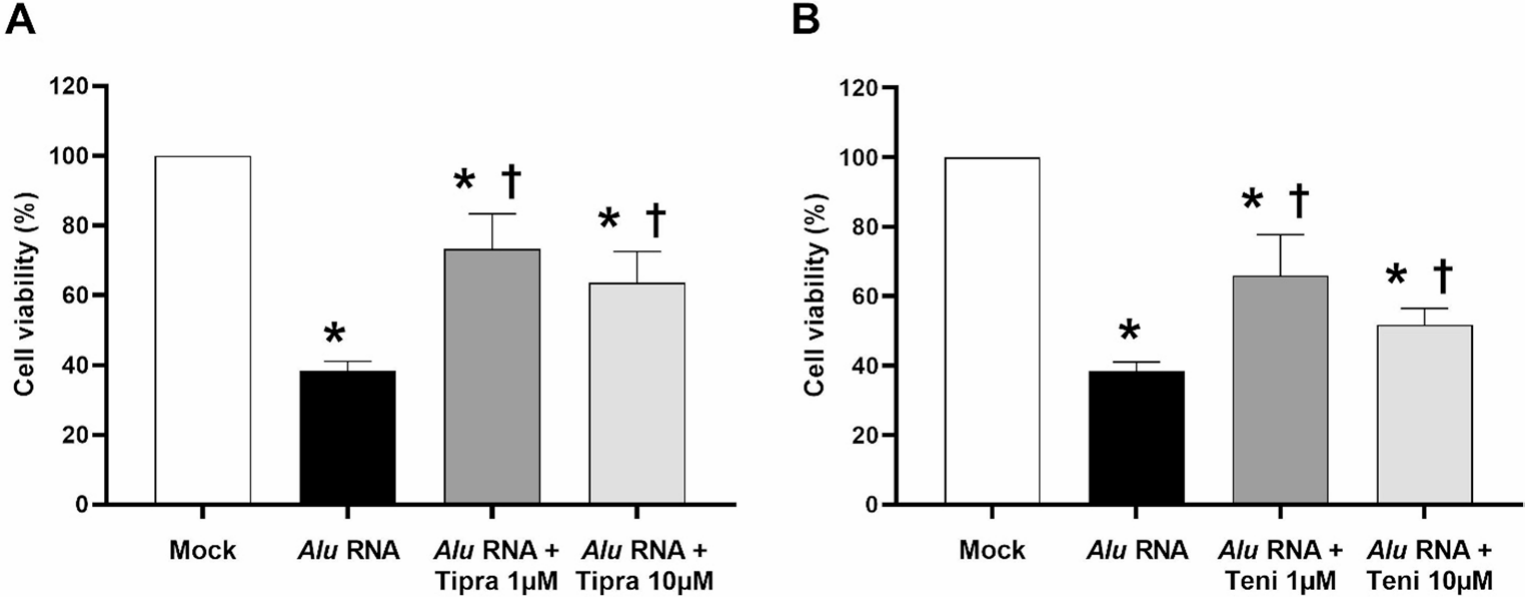
Tipranavir and teniposide rescued ***Alu*** RNA-induced hRPE cytotoxicity. hRPEs cells were pre-treated with tipranavir (Tipra; 1 and 10 µM, **A**), teniposide (Teni,1 and 10 µM, **B**) and vehicle (Mock, 0.5% DMSO) for 1 h before the transfection with *Alu* RNA. Cell viability was evaluated 24 h later by the MTS assay. Values are reported as mean percentages ± SD (*n* = 4) over non-transfected cells (Mock, 100%). *p < 0.05 vs. Mock, ^†^*p* < 0.05 vs. *Alu* RNA

Since it has been demonstrated that *Alu* RNA induced cell death in human RPE cells (hRPEs) through the activation of NLRP3 inflammasome activation via P2X7R [33, 34], we investigated whether Tipra and Teni could protect hRPEs from *Alu* RNA-mediated signaling. Firstly, we assessed whether these two compounds could rescue *Alu* RNA-induced cytotoxicity. For this purpose, hRPEs were treated with drugs or with vehicle (0.5% DMSO) for 1 h and then transfected with in vitro transcribed *Alu* RNA. After 24 h, cell viability was measured using the MTS assay. As shown in Fig. 7, pre-treatment with Tipra (Fig. 7A) and Teni (Fig. 7B) at 1µM and 10µM concentration significantly rescued *Alu* RNA-induced cytotoxicity (*p* < 0.05).

**Fig. 8:**
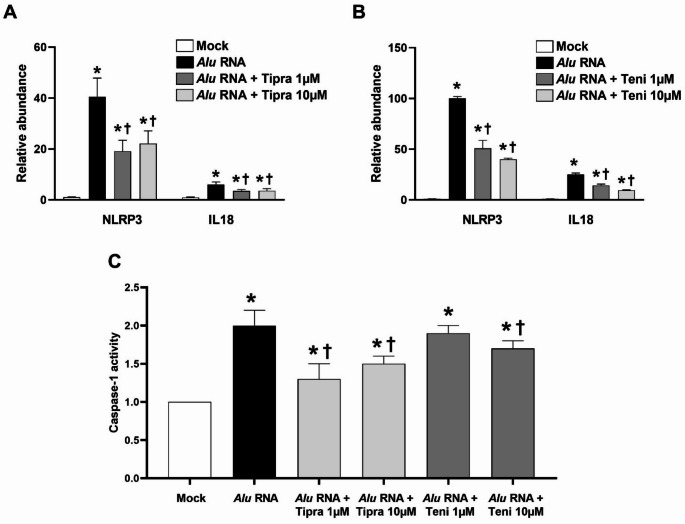
Tipranavir and teniposide rescued the ability of ***Alu*** RNA to prime and activate the NLRP3 inflammasome in hRPEs. hRPEs were pre-treated with different concentrations (1 µM and 10 µM) of tipranavir (Tipra; panel **A**), teniposide (Teni; panel **B**) and vehicle (0.5%, DMSO) for 1 h before the transfection with *Alu* RNA. NLRP3 and IL18 mRNA expression was evaluated by qRT-PCR. Values are expressed as mean percentages ± SD over non-transfected cells (Mock, 100%). *p < 0.05 vs. Mock, †*p* < 0.05. **C**) Quantification of Caspase-1 activity was measured by flow cytometry (Fig. 2S). Values are reported as fold-increase ± SD (*n* = 4) over non-transfected cells (Mock, 1). *p < 0.05 vs. Mock, ^†^*p* < 0.05 vs. *Alu* RNA

The induction of NLRP3 inflammasome by *Alu* RNA in hRPEs requires two signals: priming and activation. Priming involves increased transcription of *NLRP3* as well as the pro-form of the inflammatory cytokine *IL18*, while activation leads to the autoproteolysis of pro-caspase-1 into its cleaved and active fragment [33]. Interestingly, we found that Tipra and Teni at 1µM and 10µM concentration significantly prevented the upregulation of *NLRP3* and *IL18* mRNAs induced by *Alu* RNA, as assessed by qRT-PCR (Fig. 8A and B). Furthermore, we analyzed the ability of these two compounds to prevent the activation of NLRP3 inflammasome by measuring the cleavage of a fluorescent substrate of Caspase-1, by using flow cytometry. As shown in Figs. 2S and 8C (Supplementary material), 1 µM and 10 µM Tipra, and 10 µM Teni, significantly (*p* < 0.05) reduced Caspase-1 activation induced by *Alu* RNA.

## Discussion

P2X7 receptor antagonists are intriguing pharmacological tools, currently under preclinical development for treatment of inflammatory and neurodegenerative diseases [8], including some retinal degenerations [1]. Interestingly, the prospective exploiting P2X7 inhibitors along with other neuroprotective strategies has been hypothesized based on two preclinical studies. The first, an in vitro study, showed an antioxidant effect in neurons co-treated with memantine (NMDA receptor antagonist) and adenosine 5’-triphosphate periodate oxidized sodium salt (oxATP, P2X7R blocker) [44]. The second, an in vivo study, demonstrated that BzATP (P2X7R agonist) reduced the antioxidant effect of memantine, suggesting a link in the signaling of these two receptors [45]. Furthermore, the role of P2X7R is currently under investigation in a clinical trial (case control study) involving Parkinson patients treated with memantine and dopamine receptor agonists, but no results have been posted or published yet (ClinicalTrials.gov - NCT03918616). P2X7R implications in glaucoma have been evidenced in several in vitro and in vivo models of the disease [46–48], and also recently by papers highlighting the role of P2X7R in retinal excitotoxicity [2] and in modulation of Müller cells activated by CD40L (a protein expressed by activated CD4^+^ T cells) [49].The involvement of the P2X7R-inflammasome pathway in DR has been largely studied in vitro and in vivo [6, 50–52]. As regards AMD, a study demonstrated the detrimental role of P2X7R receptor in geographic atrophy, the early non-proliferative stage of AMD [34], and retinas of P2X7R KO mice were protected by oxidative stress [53]. Intriguingly, Drysdale et al. (2022) showed that monocytes of late-stage AMD patients highly express P2X7R [4]. Thereby, the interest in P2X7R as a crucial pharmacological target for treatment of inflammatory retinal diseases is increasing.

As largely discussed by Di Virgilio et al. in 2017 [12], besides the intrinsic attrition of clinical trials, clinical development of allosteric antagonists of P2X7R could be hampered by specific structural characteristics of the receptor itself, which shows a narrowed site for allosteric antagonists in the open state, i.e. in presence of high ATP concentration, as it happens at site of inflammation or during pyroptosis [13].

Overall, recent structural data of P2X7R have shown a high grade of functional complexity based not only on ATP concentration, but also on lipid composition of cell membrane [54] and on post-translational modifications such as channel palmytoilation [14]. Unfortunately, the search of novel ligands for P2X7R, rational drug design of drugs, with the support of in silico approaches [17, 55, 56], cannot consider these structural and then functional complexities. Indeed, according to the latest structural findings [14] and virtual screening protocols [56], we employed for virtual screening our previously updated model of full length hP2X7R [17], using as template the apo full-length structure of rat P2X7R [14]. The choice of apo structure has been justified by the findings of Karasawa and Kawate 2016 [13], reporting low accessibility of allosteric antagonists in the ATP-P2X7R structure.

We applied a virtual screening protocol that was found to be successful in drug repurposing of FDA approved drugs [57]; this protocol within the Schrödinger Maestro environment employs increasing precision molecular docking steps, and final MM-GBSA calculations. We identified six compounds, over ~ 10,000 FDA approved drugs, as putative P2X7R ligands: tipranavir; bazedoxifene; atracurium besylate; teniposide; etoposide; nelfinavir. Among these six compounds, tipranavir and nelfinavir are non-analog anti-HIV drugs, not structurally related to zidovudine. Zidovudine was previously reported to be an allosteric P2X7R antagonist, inhibiting Ca^2+^ flux and EtBr uptake on mioblasts [15], even though the activity of zidovudine was not assessed through selective and specific binding assays, such as the assays we hereby used according to a previous published protocol [56]. However, neither virtual screening nor our specific ligand binding assay (with two different assays on human P2X7R) revealed for zidovudine an activity on P2X7R (Tables 1, 2 and 3). Besides that, a recent retrospective clinical study highlighted that the prevalence of AMD signs or retinopathy in HIV patients treated with antiviral drugs, such as zidovudine, was not significantly different from prevalence in naïve HIV subject, thereby, the authors concluded that any sign of retinopathy would not be ascribed P2X7R antagonist activity of antiviral drugs [58].

Ligand binding study, using as P2X7R selective ligands references BzATP (selective agonist) and A438079 (selective antagonist) univocally determined the mechanism of action of repurposed compounds, bazedoxifene, tipranavir and teniposide, along with their IC50 as P2X7R antagonists. Involvement of P2X7 receptor in retinal diseases [2, 34, 50, 59] along with increase of ATP levels with hypoxia, high glucose or Alu-RNA induced damage has been already reported [33, 60–63]. Tipranavir and teniposide protected retinal cells from different stimuli that partially resemble pathogenic mechanisms of glaucoma, DR and AMD. Main limitation of our study is lack of in-depth analysis of complex pathways linked to P27R inhibition, such as evidenced in our previous in vitro and in vivo study [50, 59]. In fact, we hereby limited our analysis in each in vitro model to evaluation of few endpoints. Obviously, only through extended proteomic and transcriptomic analysis the weight of pathways other than P2X7R signaling would be revealed [64].

Our binding studies (Tables 3 and 4 ) have showed another element of attrition in development of P2X7R ligands, i.e. different species selectivity of ligands [65]. Very recently, a paper evidenced that P27X orthologs (human, mouse and rat) have shown different steric hindrance in orthosteric and allosteric sites of the receptor. Specifically, the hP2X7 allosteric pocket is larger than mouse and rat pockets, due to Val312 residue that forces different rotameric conferomations of other receptor residues [66]. This difference may contribute to difference in species selectivity, that we found in our binding experiments. In fact, compounds retrieved with virtual screening were found to be inactive at rat P2X7R channel, while only atracurium besylate and tipranavir were found to be active at mice P2X7R. Meanwhile bazedoxifene, tipranavir and teniposide have been identified by binding assays to be human P2XR antagonists (Tables 3 and 4). Indeed, our structural model was successful for identification of antagonists for hP2XR, and partially for mice P2X7R, confirming species selectivity, which should be considered also in preclinical in vivo models of diseases, when testing for ligands efficacy. We can infer that bazedoxifene, tipranavir and teniposide would be allosteric P2X7R antagonists because they were screened in the allosteric pocket of hP2X7R updated model and tested for their binding activity along the positive control P2X7R allosteric antagonist A438079 [41].

Overall, our findings strengthen the need for ligand binding over different P2X7R species, after optimization of structural models and protocols for structure-based drug design approaches, that need further implementation of species selectivity [6, 17, 50, 56, 65].

JNJ47965567 (7.7 median pIC50) and A438079 (6.9 median pIC50) are potent allosteric antagonists at hP2X7R with IC50 at nanomolar range. As regards as our screening campaign and binding studies of drugs active as P2X7R antagonists, bazedoxifene, tipranavir and teniposide showed micromolar activity, thereby these compounds are not potent P2X7 inhibitors, but structures would be optimized to improve their pharmacodynamic properties.

Several reports have shown anti-inflammatory effects of bazedoxifene in different experimental settings [46–50]. Worthy of note, we have found, for the first time, a P2X7R antagonistic activity for bazedoxifene and tipranavir. However, we have not tested bazedoxifene in our in vitro models of glaucoma, DR and AMD due to its cell toxicity on exposed retinal cells. As regards as teniposide, it is currently clinically tested for treatment of retinoblastoma [26]. Two reports have shown that teniposide would be a hP2X7R antagonist [67], bearing also an anti-inflammatory and anti-nociceptive activity [23].

Teniposide and tipranavir protected Müller cells from hypoxia damage as model of glaucoma, modulating expression of HIF-1α [29, 30]. HIF-1α plays a crucial role in inflammation by activating NF-κB pathway, with the following transcription of pro-inflammatory mediators (i.e. IL-1β, TNF-α) [68].

Furthermore, teniposide and tipranavir showed anti-inflammatory and antiangiogenic effect in our in vitro models of DR [31, 32], also reducing VEGF-A. Indeed, VEGF-A represents a key protein overexpressed in acute and chronic inflammation, promoting retinal neovascularization [69]. In vivo studies would confirm our in vitro results, but these studies should take into account several factors such as: translational impact of in vivo models, teniposide and tipranavir P2X7R ortholog selectivity, retinal bioavailability after drug administration, along with development of nanotechnological topical ocular formulation of repurposed drugs [70].

Finally, tipranavir and teniposide exerted protective effects on hRPEs challenged with *Alu* RNA [33], inhibiting inflammasome activation, inflammatory priming, mature inflammatory cytokines release, and caspase-1 activation, with a mechanism linked, at least in part, to the P2 × 7R antagonism.

## Conclusions

A new mechanism of action of bazedoxifene, tipranavir and teniposide was discovered through specific P27R binding assays. These drugs are hP2X7R antagonists (micromolar IC50), whose scaffold can be exploited to design novel anti-inflammatory ligands. However, bazedoxifene was not well tolerated by retinal cells, and at lower concentrations would not be active at P2X7R. In conclusion, leveraging from the findings generated in the present study, tipranavir and teniposide are worthy of further investigation to explore their therapeutic profile to manage retinal inflammatory degenerative conditions.

## Supplementary Information

Below is the link to the electronic supplementary material.

Supplementary Material 1 (PPTX 11.1 MB)

Supplementary Material 2 (DOCX 3.09 MB)

## Data Availability

Raw data will be provided by the corresponding author under request.
