# Supplementary material for: Effects of New P2X7R Antagonists on Retinal Inflammatory Degenerative Conditions
Source: Inflammation. 2026 Apr 25;49(1):151. doi: 10.1007/s10753-026-02513-7 (PMC13253766; doi:10.1007/s10753-026-02513-7)
Supplement: Supplementary file 1 — Supplementary Material 1 (PPTX 11.1 MB) [file 10753_2026_2513_MOESM1_ESM.pptx]

## Slide 1
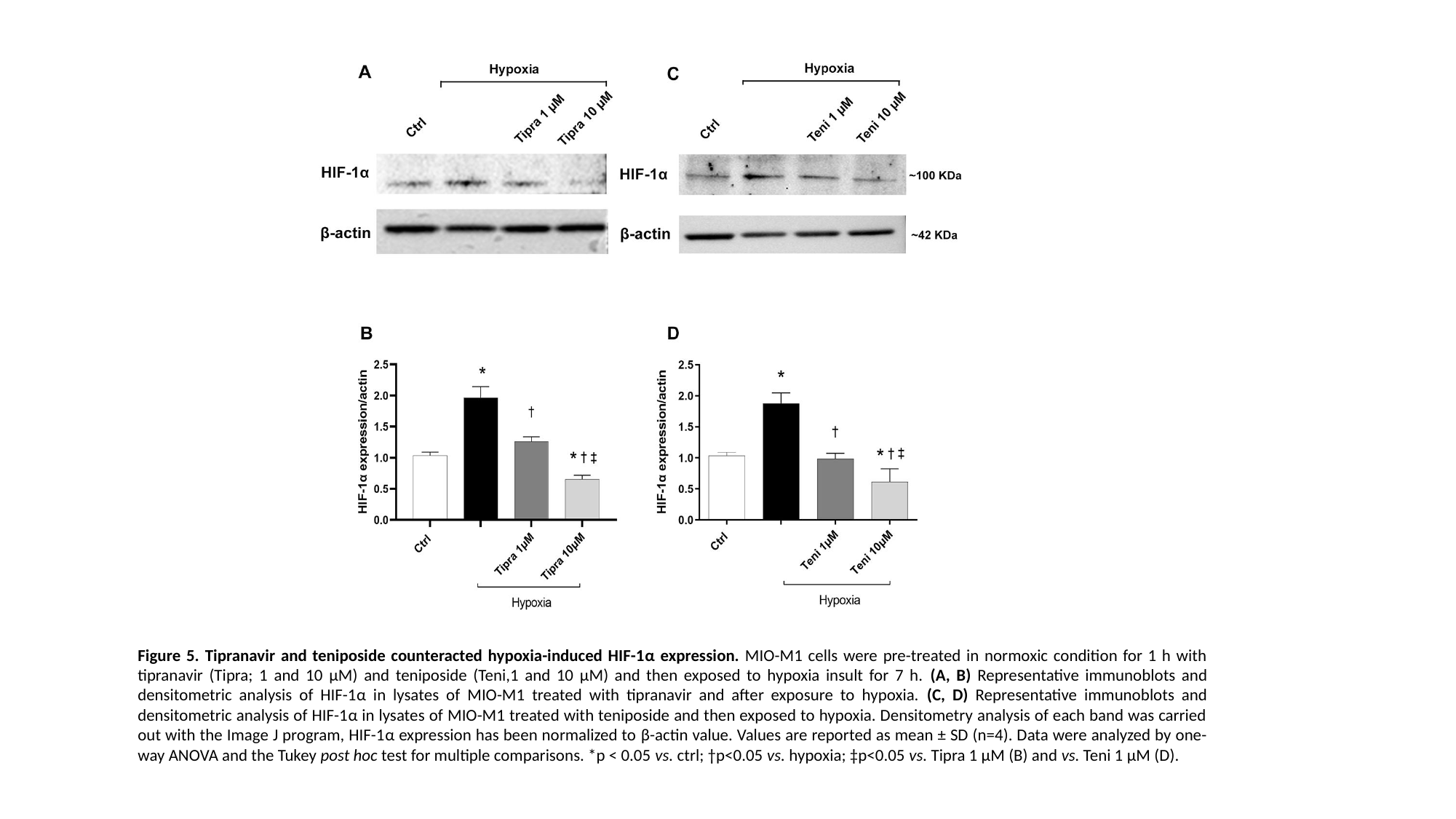

Figure 5. Tipranavir and teniposide counteracted hypoxia-induced HIF-1α expression. MIO-M1 cells were pre-treated in normoxic condition for 1 h with tipranavir (Tipra; 1 and 10 µM) and teniposide (Teni,1 and 10 µM) and then exposed to hypoxia insult for 7 h. (A, B) Representative immunoblots and densitometric analysis of HIF-1α in lysates of MIO-M1 treated with tipranavir and after exposure to hypoxia. (C, D) Representative immunoblots and densitometric analysis of HIF-1α in lysates of MIO-M1 treated with teniposide and then exposed to hypoxia. Densitometry analysis of each band was carried out with the Image J program, HIF-1α expression has been normalized to β-actin value. Values are reported as mean ± SD (n=4). Data were analyzed by one-way ANOVA and the Tukey post hoc test for multiple comparisons. *p < 0.05 vs. ctrl; †p<0.05 vs. hypoxia; ‡p<0.05 vs. Tipra 1 µM (B) and vs. Teni 1 µM (D).

## Slide 2
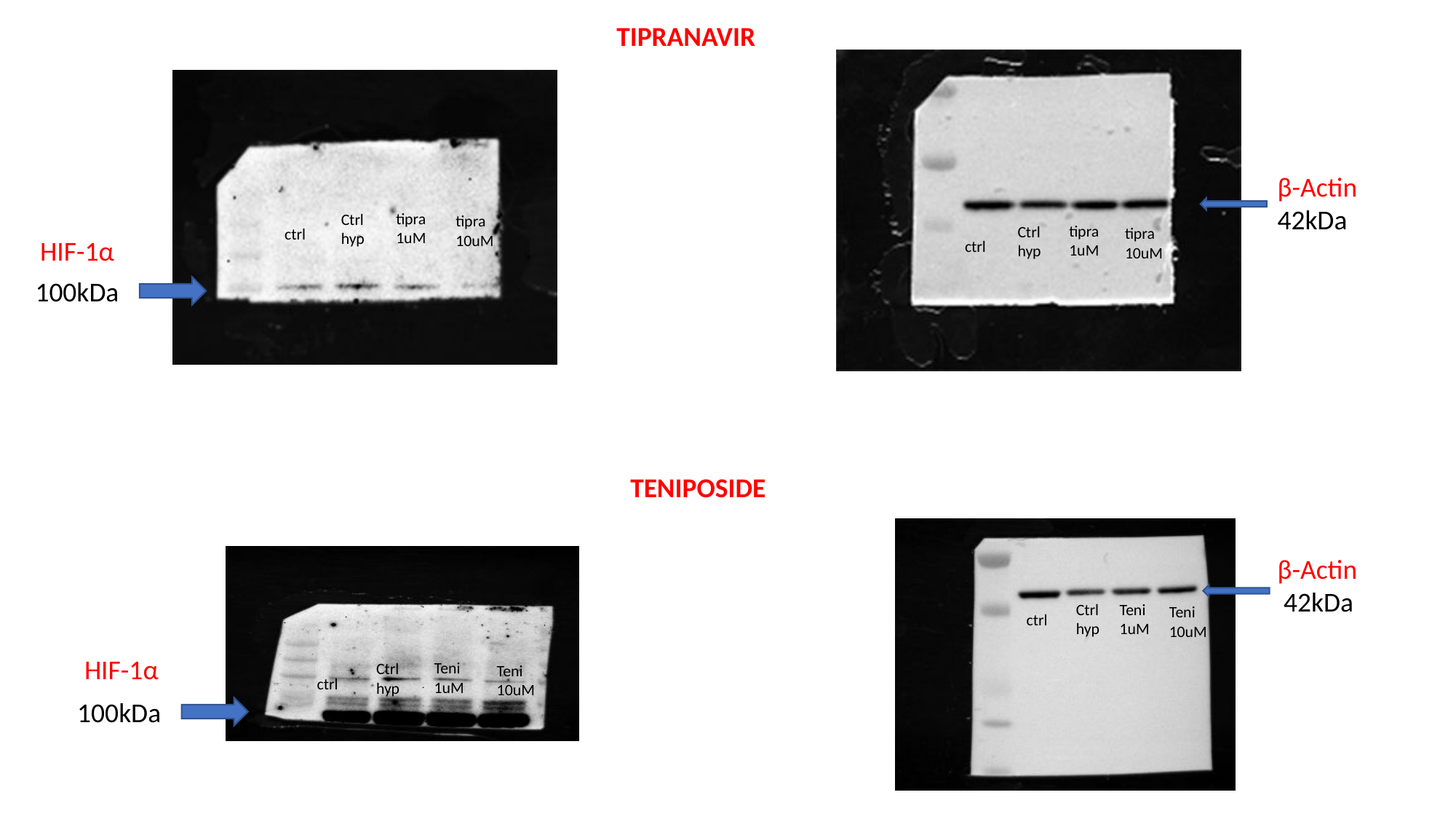

TIPRANAVIR
β-Actin 42kDa
tipra 1uM
Ctrl
hyp
tipra 10uM
ctrl
tipra 1uM
Ctrl
hyp
tipra 10uM
ctrl
HIF-1α
100kDa
TENIPOSIDE
100kDa
Teni 1uM
Ctrl
hyp
Teni 10uM
ctrl
β-Actin
 42kDa
Ctrl
hyp
Teni 1uM
Teni 10uM
ctrl
HIF-1α

## Slide 3
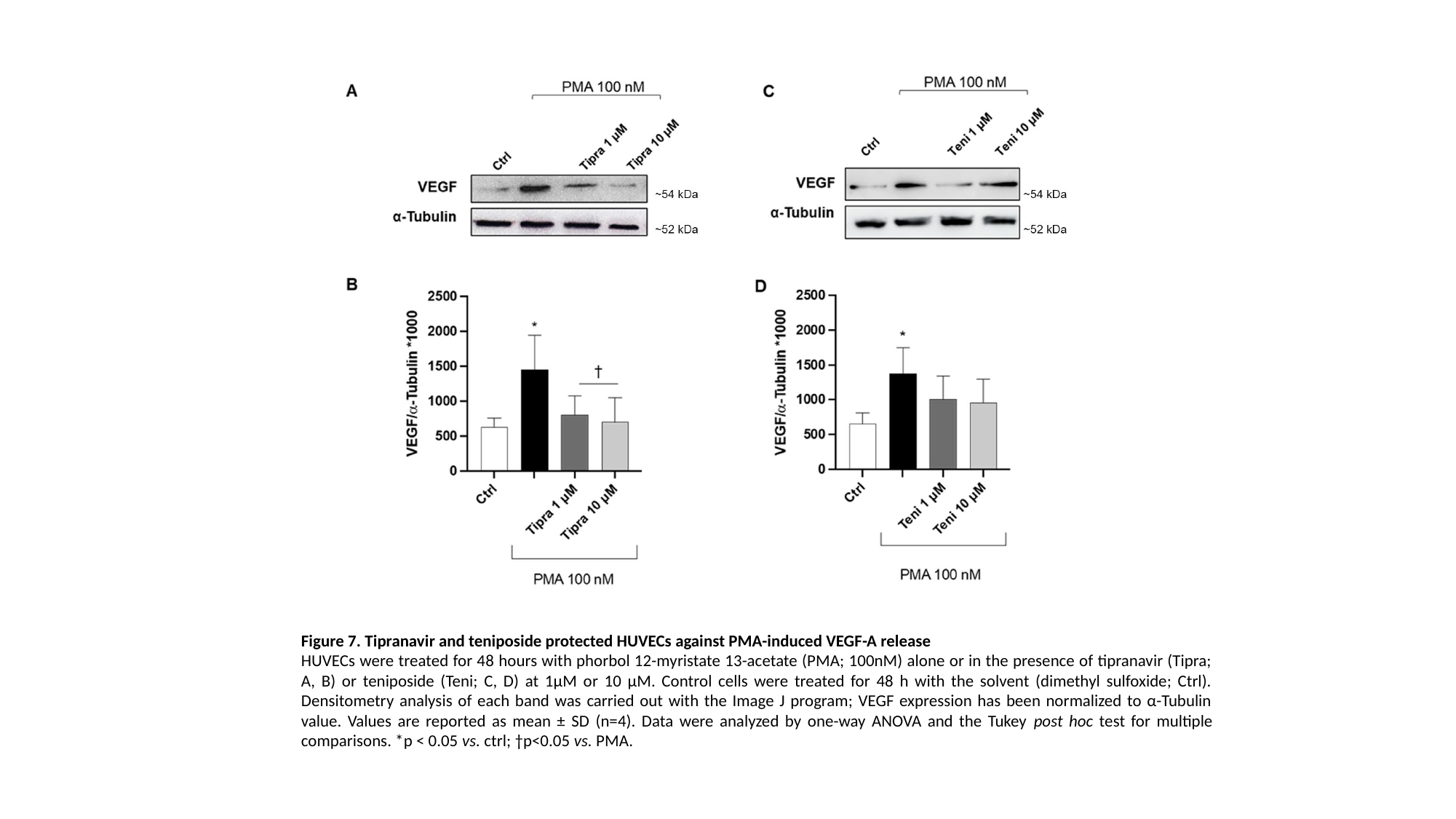

Figure 7. Tipranavir and teniposide protected HUVECs against PMA-induced VEGF-A release
HUVECs were treated for 48 hours with phorbol 12-myristate 13-acetate (PMA; 100nM) alone or in the presence of tipranavir (Tipra; A, B) or teniposide (Teni; C, D) at 1µM or 10 µM. Control cells were treated for 48 h with the solvent (dimethyl sulfoxide; Ctrl). Densitometry analysis of each band was carried out with the Image J program; VEGF expression has been normalized to α-Tubulin value. Values are reported as mean ± SD (n=4). Data were analyzed by one-way ANOVA and the Tukey post hoc test for multiple comparisons. *p < 0.05 vs. ctrl; †p<0.05 vs. PMA.

## Slide 4
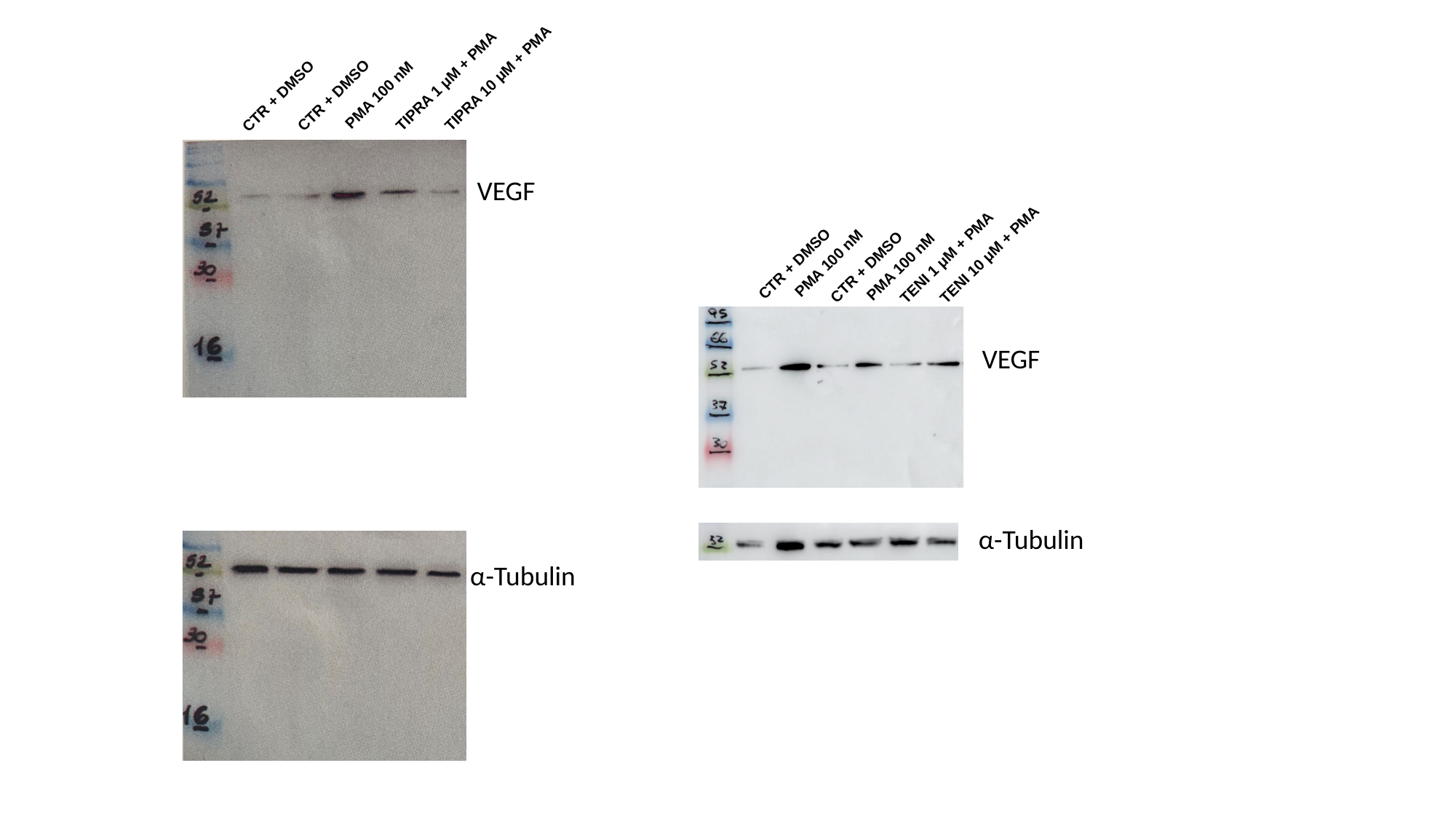

TIPRA 10 μM + PMA
TIPRA 1 μM + PMA
PMA 100 nM
CTR + DMSO
CTR + DMSO
VEGF
TENI 10 μM + PMA
TENI 1 μM + PMA
PMA 100 nM
CTR + DMSO
PMA 100 nM
CTR + DMSO
VEGF
α-Tubulin
α-Tubulin
