# Supplementary material for: Effects of New P2X7R Antagonists on Retinal Inflammatory Degenerative Conditions
Source: Inflammation. 2026 Apr 25;49(1):151. doi: 10.1007/s10753-026-02513-7 (PMC13253766; doi:10.1007/s10753-026-02513-7)
Supplement: Supplementary file 2 — Supplementary Material 2 (DOCX 3.09 MB) [file 10753_2026_2513_MOESM2_ESM.docx]

**Effects of new P2X7R antagonists on retinal inflammatory degenerative conditions**

Chiara Bianca Maria Platania^1,2*^, Federica Conti^1*^, Maria Consiglia Trotta^3*^, Nicoletta Marchesi^4^, Sonia Panico^5^, Francesca Lazzara^2,6#^, Caterina Claudia Lepre^3^, Marina Russo^3,7^, Carlo Gesualdo^8^, Francesca Simonelli^8^, Michele D’Amico^3^, Filippo Drago ^1,2^, Alessia Pascale^4^, Settimio Rossi^8^, Valeria Tarallo^5^ and Claudio Bucolo ^1,2^.

^1^Department of Biomedical and Biotechnological Sciences, University of Catania, Catania, Italy;^2^Center for Research in Ocular Pharmacology-CERFO, University of Catania, Catania, Italy;^3^Department of Experimental Medicine, University of Campania "Luigi Vanvitelli", Naples, Italy;^4^Department of Drug Sciences, Section of Pharmacology, University of Pavia, Pavia, Italy;^5^Angiogenesis Lab, Institute of Genetics and Biophysics ‘Adriano Buzzati-Traverso’ - CNR, Naples, Italy;^6^Department of Medicine and Surgery, "Kore" University of Enna, 94100 Enna, Italy;^7^Department of Mental, Physical Health and Preventive Medicine, University of Campania "Luigi Vanvitelli", Naples, Italy;^8^Multidisciplinary Department of Medical, Surgical and Dental Sciences, University of Campania "Luigi Vanvitelli", Naples, Italy

*equal contribution

# correspondence to Francesca Lazzara: [francesca.lazzara@unikore.it](mailto:francesca.lazzara@unikore.it)


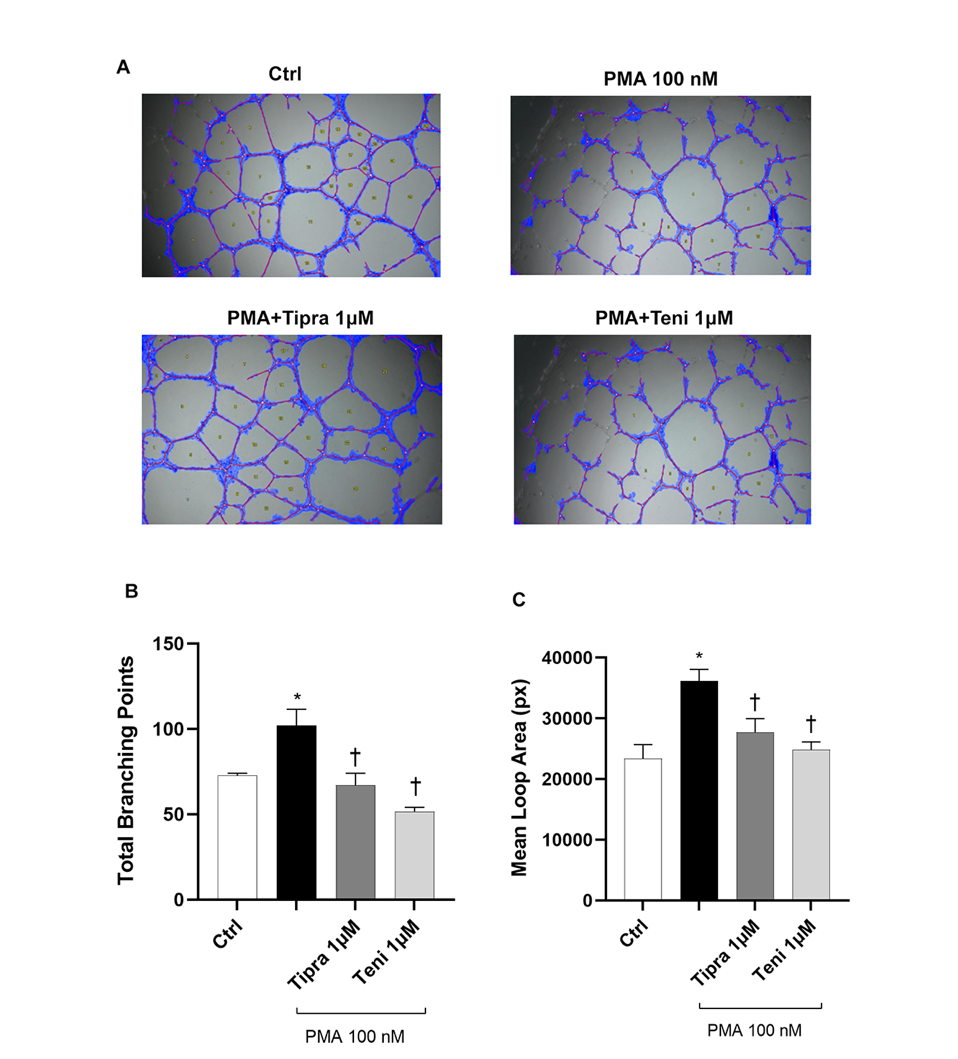


**Figure 1S: Effect of tipranavir and teniposide on angiogenesis.** HUVECs cells were pre-treated for 1 h with tipranavir (Tipra 1 µM) and teniposide (Teni 1 µM) and then challenged for 48 hours with phorbol 12-myristate 13-acetate (PMA; 100nM) alone or in the presence of tipranavir or teniposide. Control cells were treated for 48 h with the solvent (dimethyl sulfoxide; Ctrl). **(A)** Representative optical phase-contrast micrographs of tubelike structures observed in the tube formation assays (Matrigel). **(B, C)** Total branching point and the mean loop area (px) of total neo-vessel network were measured using WimTube (https://www.wimasis.com/en/WimTube, Wimasis GmbH, Munich, Germany).

Values are reported as mean ± SD (n=4). Data were analyzed by one-way ANOVA and the Tukey *post hoc* test for multiple comparisons. *p < 0.05 *vs.* ctrl; †p<0.05 *vs.* PMA.


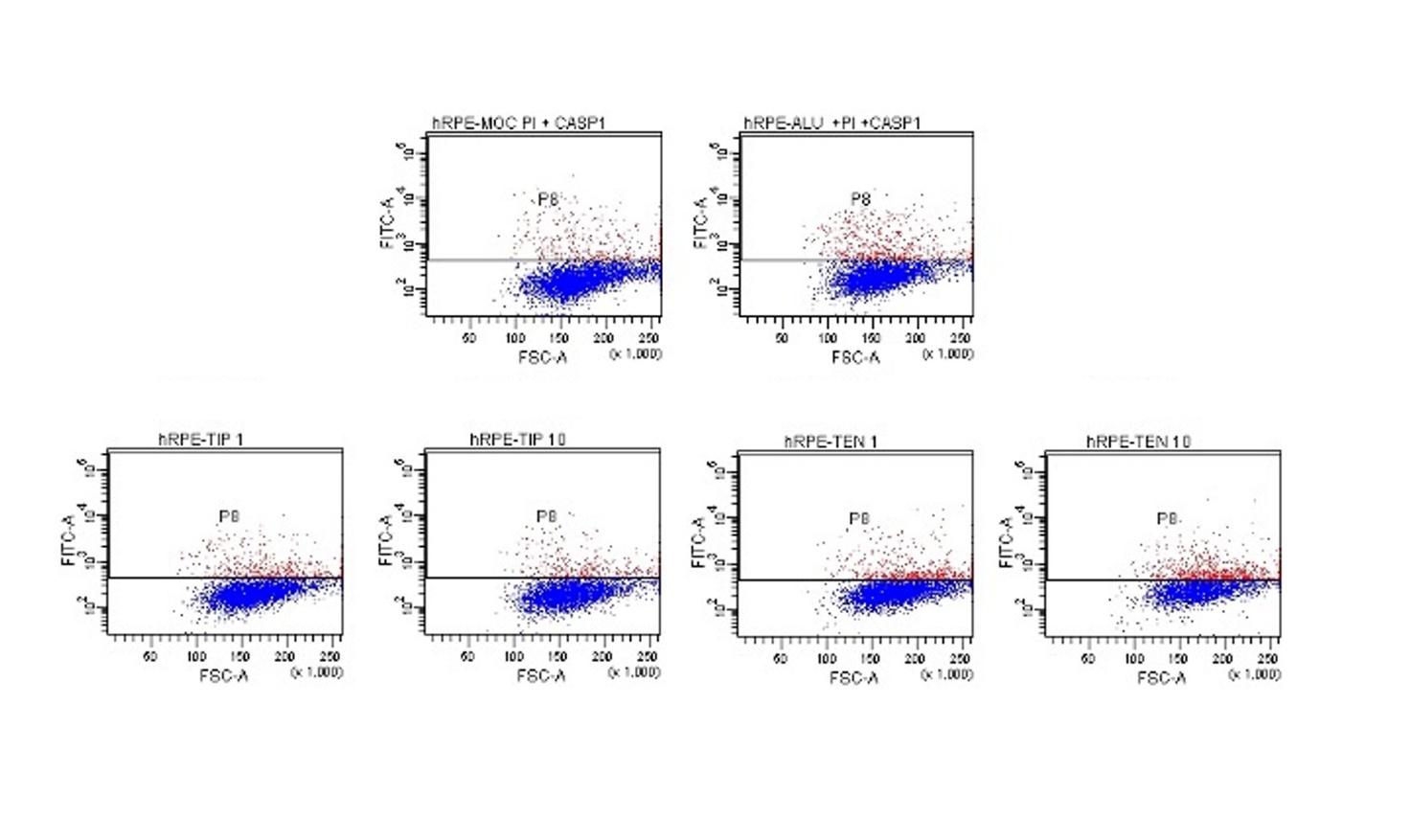


**Figure 2S: Effect of tipranavir and teniposide on Caspase-1 activation.** Representative flow cytometry plots showing increased fluorescence intensity of Caspase-1 activity in hRPEs pre-treated with different concentrations (1µM and 10µM) of tipranavir (Tip) or teniposide (Ten) or vehicle (DMSO) and then transfected with *Alu* RNA as compared with control cells (MOCK).
